# Intrinsic Capacity Across 15 Countries in the Survey of Health, Aging, and Retirement in Europe

**DOI:** 10.1001/jamanetworkopen.2025.9792

**Published:** 2025-05-12

**Authors:** Meimei Chen, Katja Hanewald, Yafei Si, Yuanyuan Gu, John R. Beard

**Affiliations:** 1 Macquarie University Centre for the Health Economy, Macquarie Business School, Sydney, Australia; 2 Australian Institute of Health Innovation, Macquarie University, Sydney, Australia; 3 School of Risk & Actuarial Studies, UNSW Business School, University of New South Wales, Sydney, Australia; 4 Australian Research Council Centre of Excellence in Population Ageing Research, Sydney, Australia; 5 Melbourne School of Population and Global Health, University of Melbourne, Melbourne, Australia; 6 Robert N. Butler Columbia Aging Center, Columbia University, New York, New York

## Abstract

**Importance:**

Intrinsic capacity (IC) is a core component of the World Health Organization’s healthy aging framework. Yet, despite multiple validations of IC across various settings, there is still a lack of longitudinal cross-national analysis.

**Objective:**

To validate the IC construct, describe variance between key demographic groups, and create population centile curves across 15 countries using data from the Survey of Health, Aging, and Retirement in Europe (SHARE).

**Design, Setting, and Participants:**

In this population-based multicenter cohort study, data from SHARE wave 5 (January to November 30, 2013) were analyzed, and subsequent care dependence in wave 6 (January to November 30, 2015) was determined. Adults 50 years and older from SHARE wave 5 with at least 1 available measure and follow-up data in SHARE wave 6 were included. Data analyses were conducted between December 11, 2022, and June 7, 2024.

**Exposure:**

SHARE waves 5 and 6.

**Main Outcomes and Measures:**

Changes in activities of daily living (ADL) and instrumental activities of daily living (IADL). Methods included structural equation modeling, bifactor analysis, and path analysis. Construct validity was tested through multiple linear regression and validity of estimates through mediation analysis. Centile curves were established using the generalized additive models for location, scale, and shape.

**Results:**

The sample included 64 872 eligible participants aged 50 to 104 years, with a mean (SD) age of 67.24 (10.01) years, of whom 35 976 (55.46%) were women. The bifactor confirmatory factor analysis model achieved good fit (comparative fit index, 0.986; Tucker-Lewis index, 0.981), suggesting an IC structure consisting of 1 general factor and 5 subdomains. Mediation analysis indicated that IC was associated with subsequent declining performance in ADL (standard coefficient [SD], −0.213 [0.002]; *P* < .001) and IADL (standard coefficient [SD], −0.209 [0.002]; *P* < .001) after adjusting for age, gender, educational attainment, socioeconomic status, and country. Socioeconomic status was associated with IC both within and between countries. Centile curves for IC by gender and country (5th, 10th, 25th, 50th, 75th, 90th, and 95th percentiles) were constructed.

**Conclusions and Relevance:**

Results of this cohort study of older adults suggest that IC was a valid and reliable measure that effectively captured individual-level aspects of functional ability. The centile curves developed during the study suggest that IC has the potential to serve as a benchmark for health status in older populations.

## Key Points

**Question:**

Is the intrinsic capacity (IC) concept of the World Health Organization a reliable and valid measure of older adults’ health and functional status across multiple countries?

**Findings:**

This cohort study of 64 872 adults 50 years and older confirmed that IC exhibits construct validity and is associated with subsequent declines in activities of daily living and instrumental activities of daily living. The study highlighted gender differences in IC and individual- and country-level economic factors and provided centile curves.

**Meaning:**

These findings suggest that IC is a valid and reliable measure that effectively captures the individual-level attributes that contribute to the functional ability of older adults, offering a benchmark for monitoring population health.

## Teaser

This cohort study determines and validates the structure of intrinsic capacity using data from participants 50 years or older from the Survey of Health, Aging, and Retirement in Europe.

## Introduction

The world is experiencing a demographic shift, with an increasing percentage of older adults in almost every population. By 2050, approximately 25% of people in regions such as Europe, Australia and New Zealand, East Asia, and North America will be older than 65 years.^1^ A better understanding of the health of this growing population segment is crucial. In the 2015 World Report on Aging and Health, the World Health Organization (WHO) proposed a framework for healthy aging focused on the development and preservation of older adults’ functional ability.^2,3^ It describes functional ability as being determined by an individual’s intrinsic capacity (IC), the environments they inhabit, and the interactions between these elements. The report defined IC as “the composite of all the physical and mental capacities that an individual can draw on.”^2^

Since the publication of the World Report, the concept of IC has gained substantial attention among clinicians and researchers as a useful measure for understanding an older adult’s health status. The WHO Integrated Care for Older People program has used IC as the entry point for care pathways.^4^

A number of studies have proposed a structure for IC that includes a summary domain and 5 subdomains: psychological, sensory, cognitive, locomotor, and vitality.^4,5,6,7,8^ This structure has utility in estimating a range of outcomes, including declines in activities of daily living (ADL) and instrumental activities of daily living (IADL),^6,7^ mortality,^9,10,11^ disease,^12^ and care dependence,^10^ even after adjustment for age, demographic characteristics, and multimorbidity. However, while 1 multicountry study used a predetermined structure to assess the prognostic power of IC in low-income and middle-income countries,^10^ to our knowledge, no study has yet statistically validated the IC construct and its predictive validity using a cross-national dataset.

In the present study, we determine and validate the structure of IC using data from the Survey of Health, Aging, and Retirement in Europe (SHARE). Our analysis is based on participants from 15 countries in SHARE wave 5 (January 1 to November 30, 2013). We first examine the structure and validity of the IC construct and then conduct a cross-national comparison of IC and subdomain scores, stratified by age, gender, country, and socioeconomic status. Last, we derive populational centile curves for IC and its subdomains, stratified by gender and country.

These are, to our knowledge, the first international level centile curves for IC, although similar efforts have been made at the national level in France.^13^ Centile curves have long been used in public health, such as for child growth charts, which monitor children’s height, weight, body mass index, and other related biomarkers against age.^14,15,16^ These centile curves enable clinicians to make decisions by comparing an individual’s health trajectory against population norms rather than simply reacting after a condition becomes apparent. Our aim is to foster the development of normative curves of IC that might enable clinicians and researchers to consider trajectories of health in older adults in much the same way as pediatricians monitor development in early life.

## Methods

### Data

SHARE is a cross-national multidisciplinary dataset offering individual-level data on health, socioeconomic, and demographic variables for individuals 50 years and older in 29 countries. Since its 2004 inception, SHARE has released 9 data waves. We used wave 5 (2013) as our baseline, as it provides the most recent and comprehensive measures for constructing IC and its 5 subdomains,^6,7^ and wave 6 (January 1 to November 30, 2015) as the follow-up. Both waves were approved by the Ethics Council of the Max Planck Society, and SHARE obtained oral consent from all participants.

We selected participants 50 years and older from SHARE wave 5 (2013) with complete age, country, and gender information, at least 1 indicator of IC, and full ADL and IADL data in both waves 5 and 6 (2015). Those with proxy-completed questionnaires, living in care homes, or with cognitive-affecting diseases were included in the baseline analysis. Spouses younger than 50 years were excluded. The final sample consisted of 64 872 participants from 15 countries (35 976 women and 28 896 men). A flowchart of the study inclusion criteria is available in eFigure 1 in Supplement 1.

The countries represented in the sample include Austria (n = 4196), Belgium (n = 5532), the Czech Republic (n = 5521), Denmark (n = 4055), Estonia (n = 5677), France (n = 4414), Germany (n = 5593), Israel (n = 2548), Italy (n = 4653), Luxembourg (n = 1585), the Netherlands (n = 4117), Slovenia (n = 2916), Spain (n = 6563), Sweden (n = 4507), and Switzerland (n = 2995). This study follows the Strengthening the Reporting of Observational Studies in Epidemiology (STROBE) reporting guideline for observational studies.

### Measures

#### Intrinsic Capacity

Building on prior research,^6,7,8^ we selected measures collected in SHARE that have the potential to provide information on IC based on 2 criteria. First, the measure should be associated with at least 1 aspect of capacity based on previous literature.^6,7,8^ Second, the measure can differentiate between high and low levels of capacity as well as detect changes in capacity within and between individuals over time.

The measures in SHARE were collected by trained interviewers and were identical in each country. The trained interviewers assessed verbal fluency, math ability, immediate and delayed memory, chair-stand test, and grip strength. Computer-assisted personal interviews determined mental health status using the EURO-D measure,^17^ and participants reported hearing and visual abilities, sleep quality, weight, weight loss, height, mobility, memory status, and concentration ability. Details are found in eAppendix 1 in Supplement 2.

### Covariates

We extracted sociodemographic and health-related covariates from SHARE wave 5, which were identified as potential confounders in previous studies.^6,7^ Our analysis included age, gender (1, men; 0, women), educational attainment (0, primary school or lower; 1, middle and high school; 2, bachelor’s degree or higher), multimorbidity, and socioeconomic status.^18^ Socioeconomic status was determined by the quartiles of annual per capita household income within the respective country of each individual (0, first quartile; 1, second quartile; 2, third quartile; 3, fourth quartile). Multimorbidity was self-reported for 20 physician-diagnosed chronic conditions (listed in eAppendix 1 in Supplement 1) and categorized as none, 1 to 2, or 3 or more chronic conditions. eTable 1 in Supplement 1 details covariate distribution by country.

### Measures of Outcome

Declining performance in ADL and IADL, based on self-reported limitations, was chosen as the outcome of interest since it provides a comprehensive measure of severe disability and loss of independence that is of clinical relevance and different from the items selected to construct IC.^6,7^ The 6 ADLs include dressing, walking across a room, bathing or showering, eating, getting in or out of bed, and using the toilet. The 7 IADLs include using a map, performing household chores or gardening, preparing a hot meal, shopping for groceries, making telephone calls, managing money, and taking medications.^17^ Declining performance in ADL and IADL is analyzed separately and is defined as participants developing at least 1 limitation in these activities between wave 5 (baseline) and wave 6 (follow-up). More details are available in eAppendix 2 in Supplement 1.

### Statistical Analysis

#### Validation

The methodology for assessing the validity of IC was conducted within the structural equation modeling (SEM) framework, similar to the approaches adopted by Beard and colleagues.^6,7^ The techniques used include confirmatory factor analysis (CFA), exploratory SEM (ESEM) framework, bifactor CFA, bifactor ESEM, and path analysis.

To evaluate the goodness-of-fit of these models, we used a range of statistical measures. These included the absolute fit index χ^2^ test, the approximate fit index root means square error of approximation (RMSEA), and the incremental fit indices, including the comparative fit index (CFI) and the Tucker-Lewis index (TLI). The weighted least square mean and variance-adjusted estimator, with robust standard errors and adjusted mean and variance, was used to assess the ESEM, CFA, and bifactor models. We assumed the missing data mechanism to be missing at random and applied pairwise deletion to handle missing data.

The construct validity was tested using linear regression, and the predictive validity was evaluated using mediation analysis.^19^ Data analyses were conducted between December 11, 2022, and June 7, 2024. More details on the methods are available in eAppendix 2 in Supplement 1.

#### Cross-Country Comparisons

We weighted and rescaled the standardized IC scores obtained from the CFA model for cross-country comparison. These scores were weighted using calibrated weights provided by SHARE to ensure the survey data were representative of the general population in the studied countries. After weighting, the scores were rescaled to a range of 0 to 100 to make them more intuitive for general readers. In this scale, 0 represents the lowest capacity among all participants, and 100 represents the highest capacity.

#### Centile Curves

To construct the centile curves, we adopted the generalized additive models for location, scale, and shape (GAMLSS) method,^20^ which is used by the WHO to construct child growth charts.^21,22^ GAMLSS was chosen because it models the mean (location), the variance (scale), and skewness or kurtosis (shape), allowing for a flexible fitting of the data.^23^ We selected the 5th, 10th, 25th, 50th, 75th, 90th, and 95th percentiles to better capture the distribution of IC scores in older adults. The statistical analyses were performed with R, version 4.2.2 (R Program for Statistical Computing).

## Results

### Validation

#### Bifactor ESEM, CFA, and Model Fit

**Figure 1.**
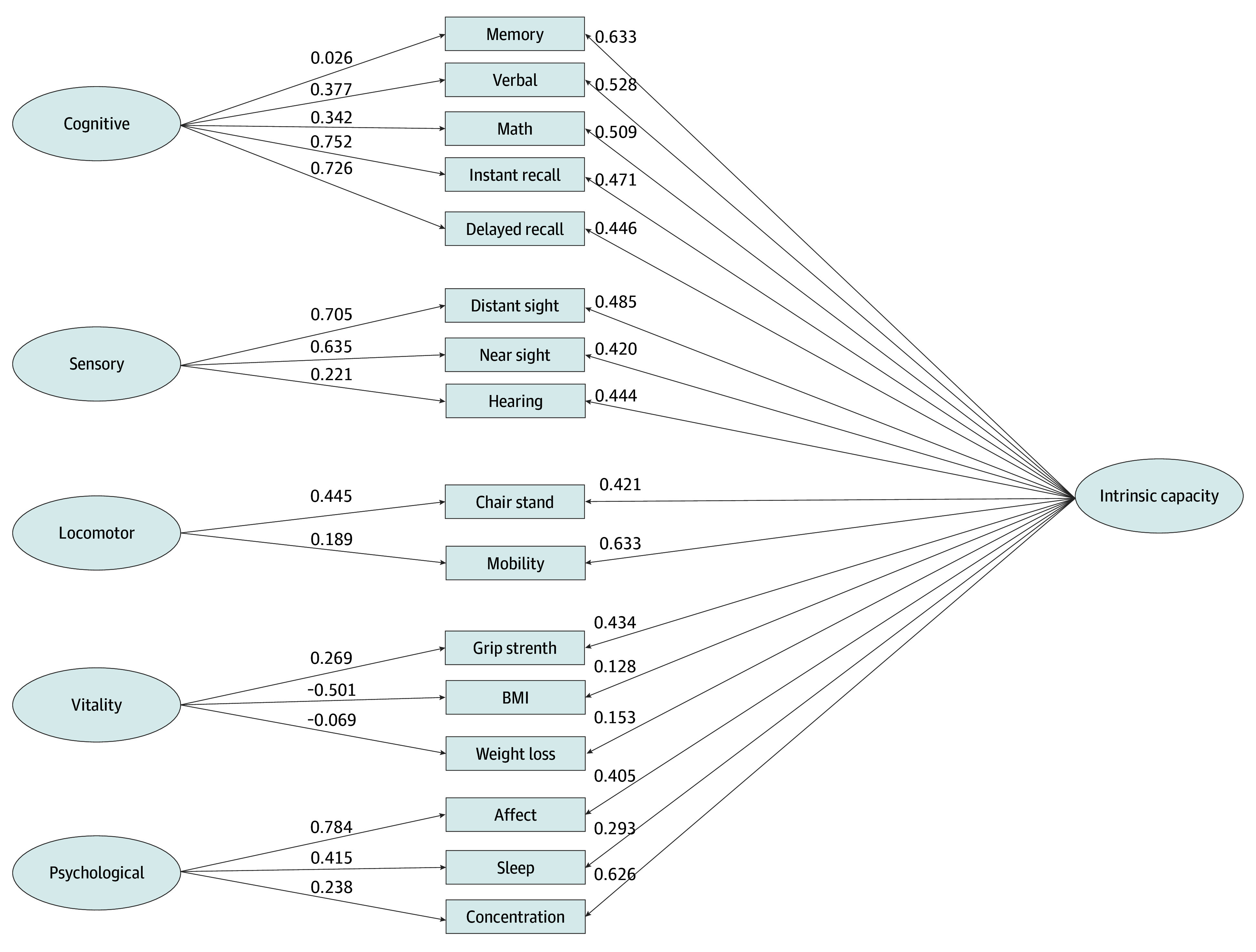
Factor Loadings of the Bifactor Confirmatory Factor Analysis Model The bifactor confirmatory factor analysis model shows the association between intrinsic capacity and its subdomains: cognitive, sensory, locomotor, vitality, and psychological. Each domain is represented by specific measurable attributes (eg, memory, hearing, mobility), with factor loadings describing their association with intrinsic capacity. Definitions for cognitive, sensory, locomotor, vitality, and psychological domains are given in eAppendix 1 in Supplement 1. BMI indicates body mass index.

The sample included 64 872 eligible participants aged 50 to 104 years, with a mean (SD) age of 67.24 (10.01) years, of whom 35 976 (55.46%) were women and 28 896 (44.54%) were men. The model fit statistics are presented in eTable 2 in Supplement 1. The 5-factor ESEM models fit well (RMSEA, 0.031; 90% CI, 0.030-0.032; CFI, 0.996; TLI, 0.991), supporting IC as a multidimensional construct. The bifactor ESEM model showed better fit (RMSEA, 0.033; 90% CI, 0.032-0.034; CFI, 0.997; TLI, 0.990), suggesting a general factor (the IC) with 5 subfactors: cognitive, sensory, vitality, locomotor, and psychological. The bifactor CFA model also fit well (RMSEA, 0.044; 90% CI, 0.043-0.045; CFI, 0.986; TLI, 0.981). We observed that most factor loadings on IC were greater than 0.3, indicating that the items contribute meaningfully to the factor (Figure 1).

#### Construct Validity

The regression analysis indicated an association between lower IC scores and factors such as higher age (eg, >80 years coefficient, −0.899; 95% CI, −0.911 to −0.867; *P* < .001), being a woman (coefficient, −0.087; 95% CI, −0.099 to −0.074; *P* < .001), lower levels of educational attainment (eg, intermediate level coefficient, 0.268; 95% CI, 0.252-0.283; *P* < .001) and income (eg, second quartile coefficient, 0.138; 95% CI, 0.121-0.156; *P* < .001), and a higher number of chronic diseases (eg, ≥3 coefficient, −0.770; 95% CI, −0.789 to −0.751; *P* < .001) (eTable 2 in Supplement 1). These findings were statistically significant after accounting for country, suggesting that each of these covariates contributed independently to the variations in IC. Considering that existing literature has established the association of these covariates with the health status of older adults, the present findings support the construct validity of IC.

The association between subdomains and covariates (eTable 2 in Supplement 1) showed that women had lower subdomain scores, except in cognitive capacity, where they scored higher (coefficient, 0.210; 95% CI, 0.192-0.228; *P* < .001). Increasing age was associated with lower scores in all subdomains, except for the psychological domain, which was higher in the group aged 60 to 69 years (coefficient, 0.071; 95% CI, 0.045-0.096; *P* < .001) than in the reference group aged 50 to 59 years. Higher educational and income levels were associated with higher subdomain scores, although the psychological score increase from the second-highest to highest income quartiles was small (coefficient, 0.214 [95% CI, 0.186-0.242] and 0.219 [95% CI, 0.189-0.248]). IC and subdomain scores significantly declined with more chronic diseases (coefficient, −0.770; 95% CI, −0.789 to −0.751; *P* < .001).

#### Pathway to Care Dependence

To identify participants with declining performance, we selected those without ADL or IADL limitations in wave 5 (2013) who developed at least 1 limitation by wave 6 (2015). Among 64 872 participants, 41 641 had no ADL limitations and complete ADL scores across waves, while 39 171 had no IADL limitations in wave 5 and complete IADL scores in wave 6. These subsets were included in the analysis. We also compared the characteristics of those lost to follow-up with those included in the final analysis for both ADL and IADL (eTable 3 in Supplement 1). Although characteristics between groups were similar, participants lost to follow-up had lower levels of educational attainment.

We first conducted a simple mediation analysis to examine the direct effect estimates of IC on ADL and IADL decline and the indirect effect estimates mediated by multimorbidity, controlling for personal characteristics (age, gender, educational attainment, income, and country). IC strongly predicted ADL and IADL decline, with dominant direct effects (standardized coefficients: −0.148 [95% CI, −0.157 to −0.140; *P* < .001] and −0.231 [95% CI, −0.246 to −0.220; *P* < .001], respectively) and minimal indirect effects via multimorbidity, accounting for only 4.5% of ADL variance (standardized coefficient, −0.007; 95% CI, −0.009 to 0.000; *P* < .001) and 2.9% of IADL variance (standardized coefficient, −0.007; 95% CI, −0.010 to 0.000; *P* < .001) (eTable 4 in Supplement 1).

Next, we applied a multilevel serial multiple mediation model to examine how wave 5 personal characteristics were associated with ADL and IADL decline in wave 6, controlling for country effects (eFigures 2 and 3 in Supplement 1). Declines in ADL and IADL were associated with both multimorbidity (standardized coefficient for ADL, 0.039 [SE, 0.002]; standardized coefficient for IADL, 0.041 [SE, 0.002]) and IC (standardized coefficient for ADL, –0.213 [SE, 0.002]; standardized coefficient for IADL, –0.209 [SE, 0.002]). IC also showed associations with age (standardized coefficient for ADL, 0.056 [SE, 0.000]; standardized coefficient for IADL, 0.050 [SE, 0.000]), gender (standardized coefficient for ADL, 0.016 [SE, 0.001]; standardized coefficient for IADL, 0.011 [SE, 0.001]), educational attainment (standardized coefficient for ADL, –0.049 [SE, 0.001]; standardized coefficient for IADL, –0.047 [SE, 0.001]), and income (standardized coefficient for ADL, –0.016 [SE, 0.000]; standardized coefficient for IADL, –0.015 [SE, 0.000]).

The pathways between personal characteristics and ADL and IADL, controlling for country effects and mediated by IC and multimorbidity, are detailed in eTable 5 in Supplement 1. Increased age and being a woman were associated with more chronic diseases, lower IC scores, and greater ADL and IADL decline. Conversely, higher educational attainment and income were associated with higher IC scores, fewer chronic diseases, and better ADL and IADL maintenance. The analysis also indicated that IC predominantly mediated effect estimates of gender, income, and educational attainment with on ADL and income and educational attainment on IADL.

### Analysis of IC Values

#### Mean IC Scores Across 15 Countries

**Figure 2.**
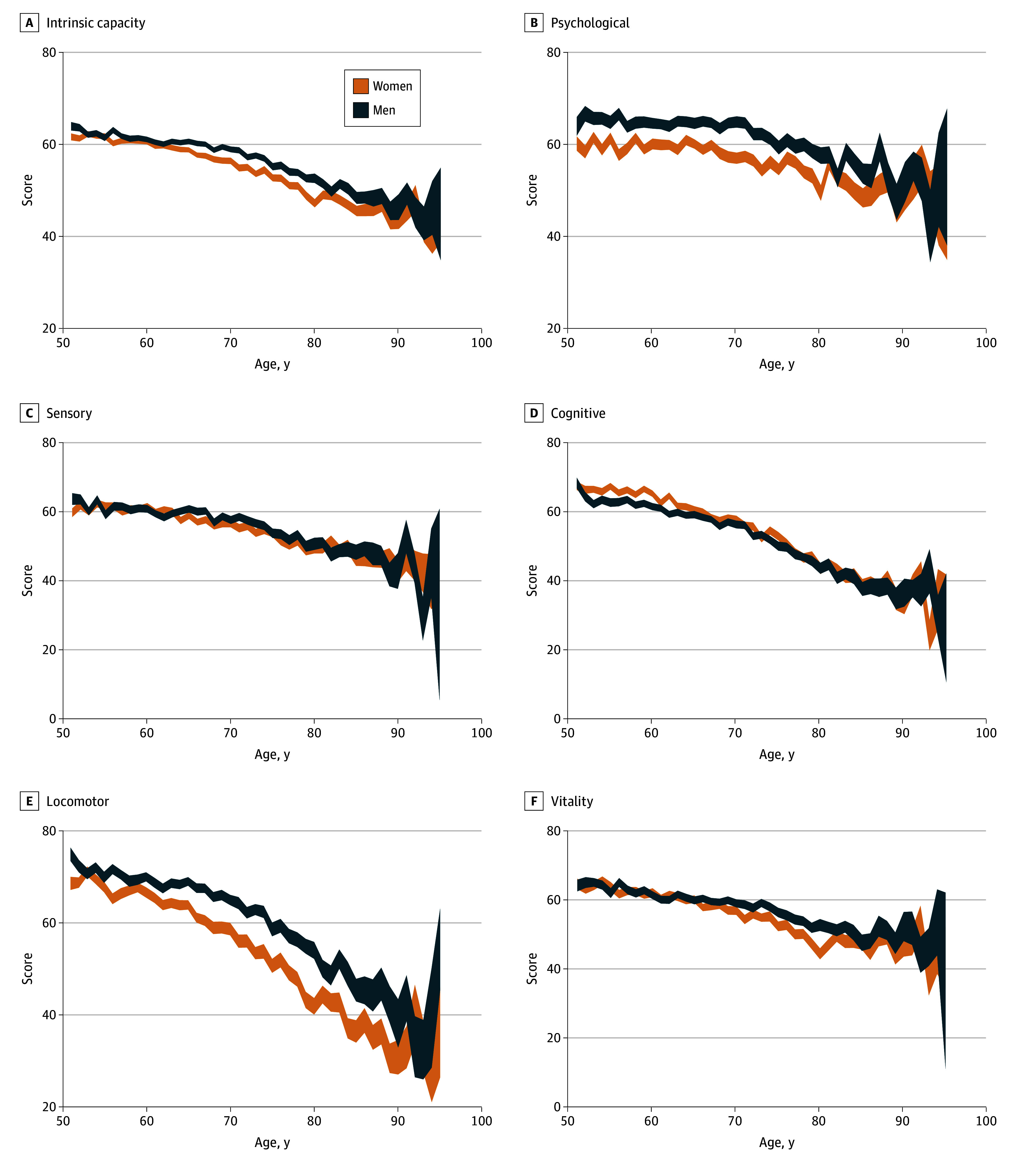
Gender Differences in Intrinsic Capacity and Cognitive, Sensory, Locomotor, Vitality, and Psychological Subdomains At each age, the mean value of the intrinsic capacity is assumed to follow a normal distribution when the number of participants is greater than 30, and a *t* distribution when the number of participants is 30 or fewer. Because of insufficient sample size, participants older than 95 years were omitted.

The analysis of this validated model, adjusted using the SHARE calibrated survey weights for national representativeness, is presented in Figure 2. A line plot displays mean IC and subdomain scores by gender across ages, with 95% CIs. On average, men scored higher on IC, with the largest gender differences in the psychological subdomain (mean [SD], 63.12 [19.63] vs 57.35 [19.90]) and locomotor subdomain (mean [SD], 65.30 [19.12] vs 58.52 [22.59]). Men also had a slight advantage in the vitality domain (mean [SD], 59.82 [18.53] vs 57.63 [20.62]) and the sensory domain (mean [SD], 58.22 [18.34] vs 56.75 [18.73]). In contrast, women younger than 70 years scored higher in the cognitive subdomain (mean [SD], 62.93 [16.92] vs 60.43 [16.44]), but scores converged between genders after this age (mean value [SD], 47.24 [18.50] for women vs 47.41 [17.32] for men).

#### Cross-Country Variations

The mean values and SDs for IC and its subdomains, categorized by gender and country, are shown in eTable 6 in Supplement 1. Men generally had higher IC scores than women across all countries, though the gender gap varied. It was narrower in countries such as Sweden (eg, IC value [SD], 60.56 [10.78] for women vs 62.09 [9.90] for men) and Denmark (eg, IC value [SD], 63.45 [11.00] for women vs 65.05 [9.73] for men) but more pronounced in Spain (eg, IC value [SD], 53.54 [11.77] for women vs 56.15 [10.70] for men) and Italy (eg, IC value [SD], 52.79 [11.81] for women vs 56.37 [10.54] for men).

**Figure 3.**
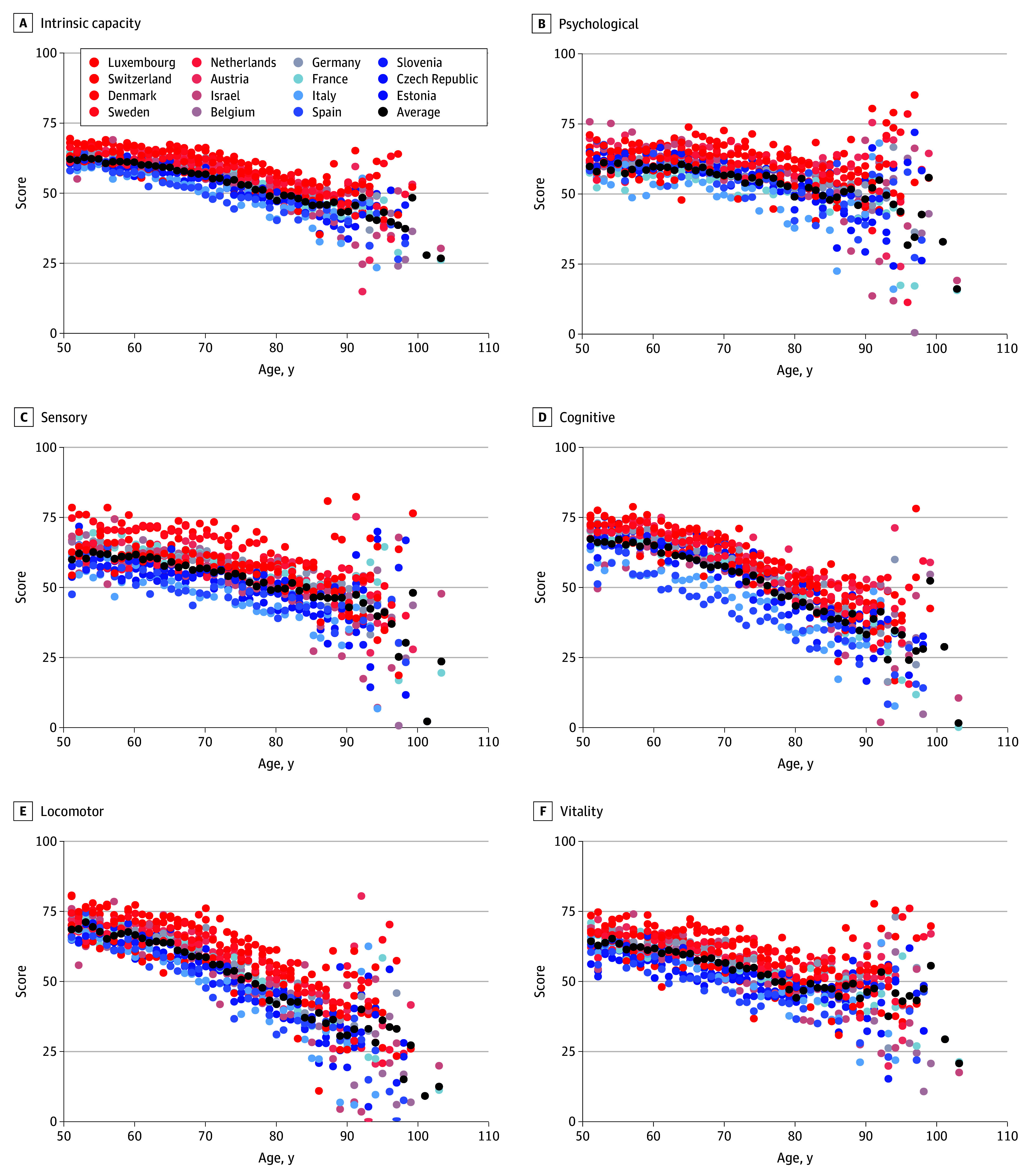
Intrinsic Capacity and Subdomain Scores Across Different Countries for Women Each point represents the mean score for IC or its subdomains for participants of a certain age across different countries. Black points indicate the overall mean values across all the countries. Countries are ordered by 2013 gross domestic product (GDP) per capita: those with a higher GDP per capita are depicted in red, while those with a lower GDP per capita are depicted in blue. GDP per capita data for the studied countries were retrieved from World Bank Group.^24^

Figure 3 illustrates IC and subdomain scores by age across countries for women, and eFigure 7 in Supplement 1 illustrates IC and subdomain scores by age across countries for men. Countries are ordered by 2013 gross domestic product (GDP) per capita presenting higher and lower GDP per capita^24^ as well as overall mean. Higher GDP per capita was generally associated with higher IC and subdomain scores (eg, Switzerland, mean [SD], 62.84 [9.84]; Denmark, 64.22 [10.44]; Slovenia, 57.47 [11.00]; and Estonia, 55.85 [10.87]).

#### IC and Socioeconomic Status

**Figure 4.**
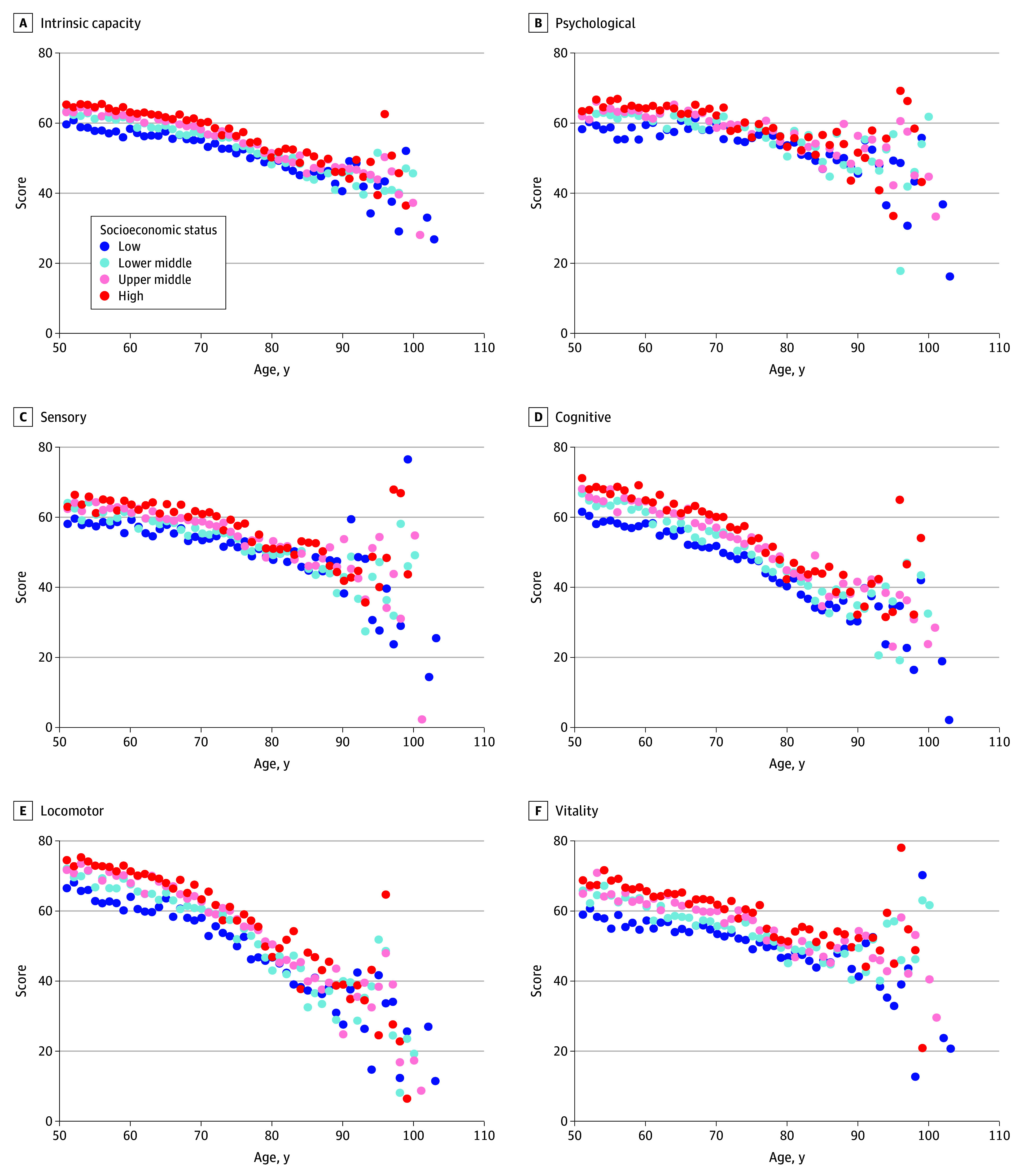
Intrinsic Capacity and Cognitive, Sensory, Locomotor, Vitality, and Psychological Subdomain Scores for Different Socioeconomic Status Groups

To examine relative socioeconomic status within each country, we divided individuals into 4 groups based on family per capita income using a quartile-based approach. Figure 4 presents a scatterplot of mean IC and subdomain scores by age and socioeconomic status, showing that higher status was associated with higher scores across all subdomains.

When analyzed by gender, women aged 50 to 70 years with higher socioeconomic status had higher IC and subdomain scores, but this advantage was not found after 70 years of age (eFigure 4 in Supplement 1). Men showed a similar pattern, though those in the highest socioeconomic group maintained their advantage until 80 years of age. Across all ages, individuals in the lowest socioeconomic group consistently had the lowest IC and subdomain scores. Mean scores by age and socioeconomic status for both genders are detailed in eTable 7 in Supplement 1.

#### Centile Curves

**Figure 5.**
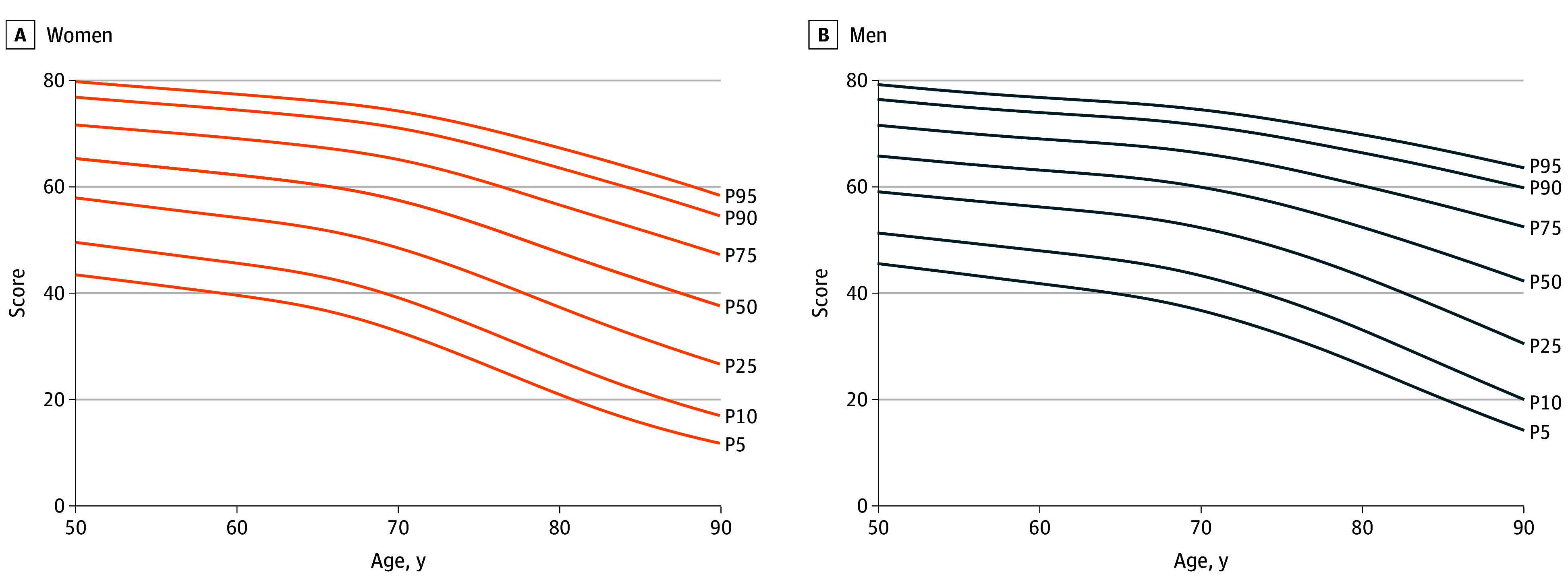
Intrinsic Capacity Across 15 Countries Smoothed centile curves for intrinsic capacity P5 indicates 5th percentile; P10, 10th; P25, 25th; P50, 50th; P75, 75th; P90, 90th; and P95, 95th.

Next, we generated population-level centile curves for IC. Figure 5 displays the smoothed centile curves for IC, while subdomain-specific curves are shown in eFigure 5 in Supplement 1. The results indicate a general decline in IC and its subdomains with age, accelerating after 70 years of age. However, unlike other subdomains, psychological scores increased between 60 and 70 years of age for both genders. Given the substantial cross-country variation in IC, we also constructed country-specific centile curves, which showed broadly similar age-related patterns but varying absolute levels across countries (eFigure 6 in Supplement 1).

## Discussion

In these representative cohorts of individuals 50 years and older from a diverse set of 15 countries in SHARE, we identified a structure for IC consisting of a general factor and 5 subdomains, which aligns with both theoretical frameworks and prior research in other settings.^6,7,8^ The structure of 1 general factor and 5 subdomains showed good construct and predictive validity, and the robustness of this approach is reinforced by the cross-country nature of our analysis.

Our longitudinal analysis confirmed the IC power to estimate declines in ADL and IADL, even after adjusting for demographic variables (age, gender, educational attainment, income, and country) and multimorbidity. This suggests that IC provides unique insights into an individual’s health and functional status beyond traditional measures such as chronological age or the presence of diseases. While more work in clinical settings is needed, tracking IC trajectories in older adults, or even from an earlier age, might help to identify those at risk of future functional decline.

Our findings reveal significant gender differences in IC and its subdomains (Figure 2). Men generally had higher IC scores than women across all age groups, with the gap widening as age increased. Within subdomains, men had higher vitality, psychological, and locomotor capacities than women. In contrast, women had higher scores on the cognitive domain than men until 70 years of age, after which the 2 genders showed similar levels (Figure 2). Patterns of sensory capacity were less clear. These findings are all generally consistent with previous research on individual outcomes^25,26,27,28,29^; however, our analysis allows each outcome to be understood in a broader functional context.

Stratification by country shows regional variations in these gender differences, with Southern European countries such as Spain and Italy displaying more pronounced differences than other regions (eTable 6 in Supplement 1). This finding aligns with those of previous studies on cognitive function across European countries.^28^ Individuals from countries with higher GDP per capita generally had higher IC scores across both genders, while, at an individual level, participants from the lowest socioeconomic status group had the lowest scores in all domains. This pattern is also consistent with previous research and highlights the importance of socioeconomic context in shaping the health of older adults.^30,31^

The centile curves for IC and its subdomains show a natural progression of IC across different ages, with a clear pattern of declining functioning with age. A similar pattern was observed in a French study conducted with participants aged between 20 and 102 years.^13^ However, we found that older adults experience accelerated declines in IC after the early 70s in women or the mid-70s in men. This contrasts with the French study, where the centile curves demonstrated a more gradual decline across all age groups.^13^ A key factor in this difference may be the observed increase in psychological subdomain scores after traditional retirement ages in our cohort.

Centile curves of IC and its subdomains have multiple potential uses. These include routine clinical assessment, where they might be used to identify individuals whose trajectories are veering away from the population norm and who may require further assessment. This is similar to the approach already taken by pediatricians to monitor child development. At a population level, the centile curves can also help compare and benchmark IC and subdomain levels against those of different settings.

### Strengths and Limitations

One strength of our study lies in the use of the SHARE dataset, which is large, representative, and longitudinal. The cross-national nature of SHARE allows consideration of the broader contextual factors that might affect our findings. However, since SHARE is not specifically designed to measure older adults’ IC and its subdomains, our study was limited to the measures available in the dataset. Consequently, we could not account for all possible factors that might be considered components of an individual’s IC. This limitation is also common in other studies, leading to inconsistencies in how IC and its subdomains have been measured. Furthermore, some items used to construct IC are self-reported, which may introduce bias into the results. Additionally, individuals who agree to participate in longitudinal studies such as SHARE may be healthier than the general population. Moreover, differences in characteristics between those included in the final mediation analysis and those lost to follow-up may introduce attrition bias, as we found that participants with lower educational levels were more likely to drop out (eTable 3 in Supplement 1). SHARE targets adults 50 years and older, so our findings may not be generalizable to younger populations, notably premenopausal women. The accelerated decline of cognitive capacity in women, but not men, after 60 years of age suggests that the impact of menopause on women’s IC scores and trajectories could be an important avenue for future research. Our data selection was also constrained by the availability of measures across different SHARE waves. We selected wave 5 as it is the most recent wave that provides all the necessary measurements to construct all 5 subdomains of IC.

Last, our findings may not be generalizable to end-of-life situations or to individuals experiencing sudden functional decline due to catastrophic events such as accidents, trauma, or rapidly progressing diseases such as aggressive cancers. Additionally, IC scores may be affected by secular trends, varying across both countries and decades due to factors such as advancements in health care, economic fluctuations, and global events such as pandemics. Cultural factors may also contribute to variations in IC scores, particularly for self-reported measures such as sleep quality, which can be shaped by societal structures and individual perceptions. Future research could explore secular trends and cultural differences using longitudinal data from early and recent waves of SHARE to better understand how IC scores evolve over time.

## Conclusions

To our knowledge, this cohort study is one of the largest and most comprehensive efforts to validate and operationalize the concept of IC. Our results have the potential to guide future research in exploring the heterogeneity of IC and its subdomains among different populations, as well as to provide tools for practical applications.

## Notes

Supplement 1. **eAppendix 1.** Measures **eTable 1.** Sample Characteristics by Country **eFigure 1.** Flowchart: Study Inclusion Criteria **eAppendix 2.** Statistical Analysis **eTable 2.** Model Fit Statistics of ESEM and CFA Models and Construct validity of IC and Subdomain Scores **eTable 3.** Comparison of Sample Characteristics Between Participants Included in the Mediation Analysis and Those Lost to Follow-Up Between Wave 5 and Wave 6 **eTable 4.** Regression Coefficients for the Direct Effects of IC on ADLs and IADLs, and Indirect Effects Through Multimorbidity **eFigure 2.** Direct and Indirect Effects of Personal Characteristics on ADL **eFigure 3.** Direct and Indirect Effects of Personal Characteristics on IADL **eTable 5.** Direct and Indirect Effects of Personal Characteristics on ADL and IADL Through Mediators (IC and Multimorbidity) **eTable 6.** Mean Values and Standard Deviations of IC and Subdomains Across Genders and Countries **eFigure 4**. Mean Values of IC and Subdomains Scores for Different Genders and Socioeconomic Status Groups **eTable 7.** Mean Value of IC and Subdomains for Participants of Different Ages, Socioeconomic Statuses, and Genders **eFigure 5**. Centile Curves for Subdomains **eFigure 6.** Centile Curves for IC in Different Countries **eFigure 7.** Intrinsic Capacity and Subdomain Scores Across Different Countries for Men
eReferences

Supplement 2.
Data Sharing Statement
