# Supplementary material for: Intrinsic Capacity Across 15 Countries in the Survey of Health, Aging, and Retirement in Europe
Source: JAMA Netw Open. 2025 May 12;8(5):e259792. doi: 10.1001/jamanetworkopen.2025.9792 (PMC12070240; doi:10.1001/jamanetworkopen.2025.9792)
Supplement: Supplement 1. — eAppendix 1. Measures eTable 1. Sample Characteristics by Country eFigure 1. Flowchart: Study Inclusion Criteria eAppendix 2. Statistical Analysis eTable 2. Model Fit Statistics of ESEM and CFA Models and Construct validity of IC and Subdomain Scores eTable 3. Comparison of Sample Characteristics Between Participants Included in the Mediation Analysis and Those Lost to Follow-Up Between Wave 5 and Wave 6 eTable 4. Regression Coefficients for the Direct Effects of IC on ADLs and IADLs, and Indirect Effects Through Multimorbidity eFigure 2. Direct and Indirect Effects of Personal Characteristics on ADL eFigure 3. Direct and Indirect Effects of Personal Characteristics on IADL eTable 5. Direct and Indirect Effects of Personal Characteristics on ADL and IADL Through Mediators (IC and Multimorbidity) eTable 6. Mean Values and Standard Deviations of IC and Subdomains Across Genders and Countries eFigure 4. Mean Values of IC and Subdomains Scores for Different Genders and Socioeconomic Status Groups eTable 7. Mean Value of IC and Subdomains for Participants of Different Ages, Socioeconomic Statuses, and Genders eFigure 5. Centile Curves for Subdomains eFigure 6. Centile Curves for IC in Different Countries eFigure 7. Intrinsic Capacity and Subdomain Scores Across Different Countries for Men eReferences [file jamanetwopen-e259792-s001.pdf]

Supplementary Online Content

Chen M, Hanewald K, Si Y, Gu Y, Beard JR. Intrinsic capacity across 15 countries in the Survey of Health, Aging, and Retirement in Europe. *JAMA Netw Open.* 2025;8(5):e259792. doi:10.1001/jamanetworkopen.2025.9792

eAppendix 1. Measures

eTable 1. Sample Characteristics by Country

eFigure 1. Flowchart: Study Inclusion Criteria

eAppendix 2. Statistical Analysis

eTable 2. Model Fit Statistics of ESEM and CFA Models and Construct validity of IC and Subdomain Scores

eTable 3. Comparison of Sample Characteristics Between Participants Included in the Mediation Analysis and Those Lost to Follow-Up Between Wave 5 and Wave 6

eTable 4. Regression Coefficients for the Direct Effects of IC on ADLs and IADLs, and Indirect Effects Through Multimorbidity

eFigure 2. Direct and Indirect Effects of Personal Characteristics on ADL

eFigure 3. Direct and Indirect Effects of Personal Characteristics on IADL

eTable 5. Direct and Indirect Effects of Personal Characteristics on ADL and IADL Through Mediators (IC and Multimorbidity)

eTable 6. Mean Values and Standard Deviations of IC and Subdomains Across Genders and Countries

eFigure 4. Mean Values of IC and Subdomains Scores for Different Genders and Socioeconomic Status Groups

eTable 7. Mean Value of IC and Subdomains for Participants of Different Ages, Socioeconomic Statuses, and Genders

eFigure 5. Centile Curves for Subdomains

eFigure 6. Centile Curves for IC in Different Countries

eFigure 7. Intrinsic Capacity and Subdomains Scores across Different Countries for Men

eReferences.

This supplementary material has been provided by the authors to give readers additional information about their work.

© 2025 Chen M et al. *JAMA Network Open*.

eAppendix 1. Measures

We note that in SHARE Wave 6, the Netherlands did not participate in the regular SHARE wave but conducted an experiment using an online survey (CAWI) or telephone interviewing (CATI) instead of face-to-face interviews (CAPI) as conducted in the rest of the SHARE Wave 6 countries, as described here: https://share-eric.eu/data/data-set-details/dutch-mixed-mode-experiment-w6-1).

Psychological

Affect: Affect was measured by the EURO-D scale within the SHARE dataset,[2] with eight specific variables (i.e., depression, pessimism, suicidality, guilt, lack of interest, irritability, lack of enjoyment, and tearfulness) designated as indicators of depressed mood. Each item was scored using a binary system, with a score of 1 assigned to the absence of the corresponding symptom and 0 assigned to its presence. The overall affect score was then calculated as the sum of the eight designated items, with higher scores indicating better affect.

Sleep: Self-reported sleep quality was evaluated using a simple dichotomous measure. Specifically, participants were asked to indicate whether they had experienced any difficulties with sleeping recently. The available response options included two categories, namely "no trouble sleeping" (scored as 1) and "trouble with sleep" (scored as 0).

Concentration: Concentration was assessed through the use of two self-reported items: concentration on entertainment and concentration on reading. Each item was scored dichotomously, with participants indicating whether they experienced any difficulties with concentration using the response options "no trouble concentrating" (scored as 1) and "having trouble concentrating" (scored as 0). To determine the overall concentration score, the responses to the two items were summed, with higher scores indicating better concentration.

Cognitive

Memory: Memory was assessed through self-report, with participants indicating their perceived level of memory using a five-point scale. Responses were categorised as poor (scored as 0), fair (scored as 1), good (scored as 2), very good (scored as 3), or excellent (scored as 4).

Verbal fluency: Verbal fluency was assessed by asking participants to verbally list as many animals as possible within a designated time frame of one minute. The corresponding score for this item was calculated based on the number of animals that were correctly named within the given time limit. In general, higher scores on this task indicate better verbal fluency.

Math: Math ability was assessed by instructing participants to perform a subtraction of 7 from 100, and to repeat this process five times in a row. The corresponding score for this item was calculated based on the number of correctly answered questions, with scores ranging from 0 to 5. Higher scores on this task are indicative of better math ability.

Recall: There are two items used to calculate recall function in SHARE Wave 5. The first one is the instant recall test: participants were asked to repeat words immediately after they heard from the examiner. The second one is delayed recall: participants were asked to repeat words they heard a while ago.

Sensory

Eyesight: Self-assessment was used to evaluate both distant and near eyesight. Participants were asked to rate their eyesight on a scale of five responses: poor, fair, good, very good, and excellent, which were scored as 0, 1, 2, 3, and 4, respectively. A higher score indicates better eyesight.

Hearing: Self-assessment was used to evaluate hearing ability, with responses rated on a scale of five levels: poor, fair, good, very good, and excellent, which were scored as 0, 1, 2, 3, and 4, respectively. A higher score indicates better hearing.

© 2025 Chen M et al. *JAMA Network Open*.

Locomotor

Chair stand: The chair stand test was performed once per participant. Participants were asked to fold their arms across their chests, stand up from a seated position on a chair and sit down again five times. The time (in seconds) taken for the participant to stand up five times without using their hands was measured using a stopwatch. Results were categorised into five levels according to ICOPE: less than 11.19 seconds (scored as 4), 11.2-13.69 seconds (scored as 3), 13.7-16.69 seconds (scored as 2), 16.7-59.9 seconds (scored as 1), and greater than 60 seconds (scored as 0).

Mobility: Participants were asked to report any difficulty they experienced when performing everyday mobility-related activities during the assessment. The activities included walking 100 metres, sitting for about two hours, getting up from a chair after sitting for long periods, climbing several flights of stairs without resting, climbing one flight of stairs without resting, stooping, kneeling, or crouching, reaching or extending arms above shoulder level, pulling or pushing large objects like a living room chair, lifting or carrying weights over 10 pounds/5 kilos, like a heavy bag of groceries, and picking up a small coin from a table. Participants were asked to indicate the number of activities that they found difficult, and a score was calculated by subtracting the number of difficult activities from 10. Therefore, higher scores indicated better mobility.

Vitality

Grip strength: Grip strength was measured using a handheld dynamometer (Smedley, S Dynamometer, TTM, Tokyo, 100 kg) on each hand. Two measurements were taken on each hand, alternating between the hands. The score was determined by the maximum grip strength recorded from either hand.

BMI: The body mass index (BMI) was calculated for all participants using their recorded weight and height. The BMI score was categorised into three levels: a BMI of less than 18.5 or greater than or equal to 30 was scored as 0, a BMI between 18.5 and 25 was scored as 2, and a BMI between 25 and 30 was scored as 1. Higher scores indicate a healthier BMI.

Weight loss: The participants were asked to report the amount of weight they had lost within the past six months. Their answers were categorised into three levels: no weight loss or less than 1 kg (scored as 2), 1-3 kg (scored as 1), and more than 3 kg (scored as 0).

Covariates

Multimorbidity: heart attack, high blood pressure, high blood cholesterol, stroke or cerebral vascular disease, diabetes or high blood sugar, chronic lung disease, cancer or malignant tumour (excluding minor skin cancers), stomach or duodenal ulcer, Parkinson's disease, cataracts, hip fracture, other fractures, Alzheimer's disease, dementia, organic brain syndrome, memory impairment, affective or emotional disorders, rheumatoid arthritis, osteoarthritis, or other rheumatism.[1]

© 2025 Chen M et al. *JAMA Network Open*.

eTable 1. Sample Characteristics by Country

Country	Women (%)	Age (SD)	Education (%)			Multimorbidity (%)			ADL (SD)	IADL (SD)
			Level 1	Level 2	Level 3	0	1 or 2	3 or more		
Austria	57.412	67.477 (9.679)	23.570	48.546	25.953	24.976	52.264	21.902	0.243 (0.89)	0.443 (1.244)
Belgium	54.935	66.245 (10.633)	37.708	26.157	32.719	21.782	51.898	26.302	0.31 (0.915)	0.446 (1.173)
Czech Republic	58.359	67.061 (9.04)	38.924	46.441	12.842	16.320	49.683	33.871	0.275 (0.883)	0.414 (1.141)
Denmark	53.539	65.407 (10.127)	19.038	38.175	41.751	24.908	49.840	25.228	0.179 (0.717)	0.309 (1.021)
Estonia	60.296	68.451 (9.722)	29.699	48.758	21.402	19.605	51.488	28.818	0.39 (1.042)	0.577 (1.336)
France	56.955	67.907 (10.411)	32.556	34.685	20.344	22.202	52.855	24.898	0.265 (0.868)	0.414 (1.165)
Germany	52.351	65.125 (10.008)	12.301	56.982	29.233	18.505	51.010	30.377	0.232 (0.832)	0.314 (1.015)
Israel	55.730	68.048 (10.209)	26.334	30.573	32.457	23.312	40.188	36.028	0.451 (1.326)	0.872 (1.778)
Italy	54.374	67.286 (9.914)	61.552	20.138	7.995	24.973	49.860	25.102	0.347 (1.136)	0.479 (1.42)
Luxembourg	52.871	64.738 (9.827)	44.479	34.196	18.549	14.196	47.950	37.855	0.249 (0.851)	0.373 (1.124)
Netherlands	55.089	66.467 (9.709)	44.596	25.043	26.864	27.812	51.931	20.233	0.153 (0.681)	0.292 (0.928)
Slovenia	56.653	67.019 (10.031)	29.012	48.320	17.044	23.731	52.846	23.422	0.219 (0.838)	0.369 (1.147)
Spain	53.741	68.665 (10.901)	53.573	9.950	9.874	19.351	50.312	30.321	0.379 (1.204)	0.64 (1.598)
Sweden	53.295	68.528 (9.346)	34.546	32.505	29.732	21.877	53.162	24.917	0.167 (0.713)	0.249 (0.894)
Switzerland	54.524	67.055 (9.746)	19.499	62.137	16.394	35.927	49.449	14.624	0.096 (0.482)	0.156 (0.611)

Notes: ADL: Activities of Daily Living; IADL: Instrumental Activities of Daily Living; SD: Standard Deviation. Sample size N = 64,872 (Women: 35 976; Men: 28 896). Missing value percentage: Education (6.3%) and Multimorbidity (0.1%). All the other variables are complete.

© 2025 Chen M et al. *JAMA Network Open*.

eFigure 1. Flowchart: Study Inclusion Criteria

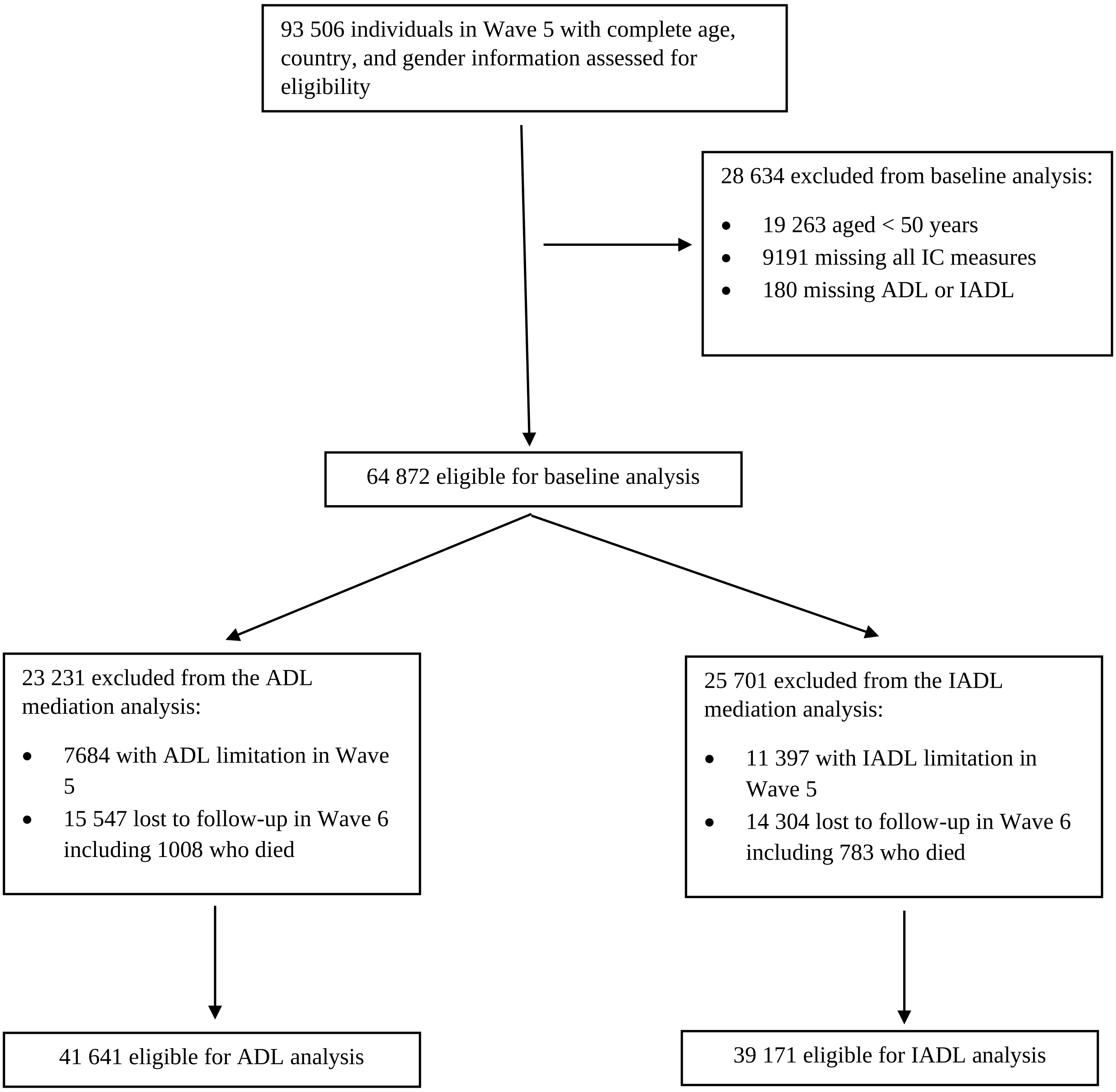

Notes: ADL: Activities of Daily Living; IADL: Instrumental Activities of Daily Living.

© 2025 Chen M et al. *JAMA Network Open*.

eAppendix 2. Statistical Analysis

Validation

We used Confirmatory Factor Analysis (CFA), Exploratory Structural Equation Modelling (ESEM) framework, bifactor CFA, bifactor ESEM, and path analysis to assess the validity of IC. A range of statistical measures were used to evaluate the goodness-of-fit of these models. These included the absolute fit index Chi-Square (χ^2), the approximate fit index root means square error of approximation (RMSEA), and the incremental fit indices, including the Comparative Fit Index (CFI) and the Tucker-Lewis Index (TLI). The Weighted Least Square Mean and Variance Adjusted (WLSMV) estimator, with robust standard errors and adjusted mean and variance, was used to assess the ESEM, CFA, and bifactor models. We assumed the missing data mechanism to be missing at random and applied pairwise deletion to handle missing data.

A series of ESEM models with varying numbers of factors as well as a bifactor ESEM were performed. The bifactor model was chosen to investigate the presence of a general IC factor with five subfactors. To evaluate the goodness-of-fit of these models, we employed a range of statistical measures. These included the absolute fit index Chi-Square (χ^2), the approximate fit index root means square error of approximation (RMSEA), and the incremental fit indices, including the Comparative Fit Index (CFI) and the Tucker-Lewis Index (TLI).

After exploring the potential structure of IC as one general factor with five subfactors, we further employed a series of CFA models to test the structure. These models included a single-factor model, a correlated five-factor model, a hierarchical (second-order) model, and a bifactor model. The CFA models were assessed using the same model fit statistics as previously described. The Weighted Least Square Mean and Variance Adjusted (WLSMV) estimator, with robust standard errors and adjusted mean and variance, was used to assess the ESEM, CFA, and bifactor models. We assumed the missing data mechanism to be missing at random and applied pairwise deletion to handle missing data.

Subsequently, we tested the construct and predictive validity of IC. Factor scores were extracted from the hierarchical CFA model using the empirical Bayes method. These factor scores have a mean of zero and a standard deviation of one. To test the construct validity, we ran a linear regression to investigate the relationship between IC factor scores and personal characteristics, including age, gender, education, income, and multimorbidity. The country location of each individual was controlled for in these regressions to account for potential unobserved confounding effects.

To test the predictive validity, we employed mediation analysis to explore the potential pathways of the relationship between baseline IC in Wave 5 (2013) and the declining performance in Wave 6 (2015). We conducted these analyses separately for ADL and IADL. Initially, we selected participants with no performance limitations (scoring 0 in ADL or IADL) in Wave 5, and then extracted their ADL or IADL scores in Wave 6. Participants who developed performance limitations in Wave 6 were assigned a score of 1, while those without limitations retained a score of 0.

We ran a mediation model to examine the direct effects of IC on declining performance in ADL and IADL, as well as the indirect effects through multimorbidity, while controlling for possible confounders (i.e., age, gender, education, income, and country). Parameter estimation was performed using the robust WLSMV estimator. Subsequently, we applied a multi-level serial multiple mediators model to investigate the influence of personal characteristics (i.e., age, gender, education, and income) from Wave 5 on subsequent performance declines in ADL and IADL in Wave 6 while controlling for countries. This method accounts for the nested data structure, where individuals are

© 2025 Chen M et al. *JAMA Network Open*.

grouped within countries. We adopted the 1-1-1 design of two-level mediation analysis to accommodate this nested structure and ensure accuracy in our analysis. In this model, variables including personal characteristics (age, gender, education, and income), IC, multimorbidity, ADL, and IADL are considered as collected at the individual level (level 1). Meanwhile, the model allows the regression slopes (i.e., the relationships between variables) to differ across countries (level 2). As a result, the model accounts for potential country-specific variations in our data. This flexibility provides a more nuanced understanding of the complex relationships between personal characteristics and health conditions while considering the differences in the countries in which individuals reside.

Cross-country comparisons

The calibrated weights in SHARE were calculated following the methodology proposed by Deville and Särndal.[3]

Centile Curves

We treated age as a continuous variable and assumed IC and subdomains followed a specific distribution selected from a range of distributions, including Box-Cox Cole and Green (BCCG), Box-Cox t (BCT), and Box-Cox Power Exponential (BCPE). In the BCCG case, GAMLSS reduces to the LMS method, which is also widely used for modelling centile curves.[4]

To select the best distribution, we used model selection metrics such as the Akaike Information Criterion (AIC) and the Bayesian Information Criterion (BIC), along with diagnostic statistics, including worm plots and Q statistics. AIC and BIC were used to compare model fit, while worm plots and Q statistics helped detect inadequacies both globally and locally. The smoothing method employed in our model is the P-splines method,[5] and the smoothing parameters (hyperparameters) were automatically tuned using build-in functions in the gamlss package in R.

© 2025 Chen M et al. *JAMA Network Open*.

eAppendix 3: Results

Validation

eTable 2. Model Fit Statistics of ESEM and CFA Models and Construct validity of IC and Subdomain Scores

Model Fit Statistics of ESEM and CFA Models

Models	Model fit statistics				
	χ^2	df	CFI	TLI	RMSEA (90% CI)
**ESEM**					
One-factor	91 305.539	104	0.888	0.870	0.116 (0.116, 0.117)
Two-factors	28 984.489	89	0.964	0.952	0.071 (0.070, 0.071)
Three-factors	9486.583	75	0.988	0.981	0.044 (0.043, 0.045)
Four-factors	5418.368	62	0.993	0.987	0.036 (0.036, 0.037)
Five-factors	3212.744	50	0.996	0.991	0.031 (0.030, 0.032)
Bifactor (one general factor and five sub-factors)[1]	2427.504	34	0.997	0.990	0.033 (0.032, 0.034)
**CFA**					
One-factor	91 305.539	104	0.888	0.870	0.116 (0.116, 0.117)
Second-factor	35 428.928	99	0.956	0.947	0.074 (0.074, 0.075)
Correlated five factors	32 545.787	94	0.960	0.949	0.073 (0.072, 0.074)
Bifactor (one general factor and five sub-factors)	11 317.921	89	0.986	0.981	0.044 (0.043, 0.045)

Note: [1]Bifactor exploratory analysis is conducted in the Exploratory Structural Equation Modelling (ESEM) framework. χ^2= Chi-square, **df** = degree of freedom, **CFI** = comparative fit index, **TLI** = Tucker–Lewis index, **RMSEA** =root mean square error of approximation, **CI** = confidence interval.

Construct validity of IC and Subdomain Scores

	**Intrinsic Capacity**	**Psychological**	**Cognitive**	**Sensory**	**Locomotor**	**Vitality**
	Coefficient (95% CI)	Coefficient (95% CI)	Coefficient (95% CI)	Coefficient (95% CI)	Coefficient (95% CI)	Coefficient (95% CI)
**Gender**						
Men	Ref	Ref	Ref	Ref	Ref	Ref
Women	-0·087 ***	-0·268 ***	0·210 ***	-0·026 **	-0·333 ***	-0·041 ***
	[-0·099, -0·074]	[-0·288, -0·248]	[0·192, 0·228]	[-0·044, -0·008]	[-0·357, -0·310]	[-0·062, -0·020]
**Age**						
50 ~ 59	Ref	Ref	Ref	Ref	Ref	Ref
60 ~ 69	-0·083 ***	0·071 ***	-0·266 ***	-0·073 ***	-0·144 ***	-0·086 ***
	[-0·099, -0·067]	[0·045, 0·096]	[-0·290, -0·243]	[-0·096, -0·050]	[-0·174, -0·114]	[-0·113, -0·060]
70 ~ 79	-0·324 ***	-0·074 ***	-0·759 ***	-0·316 ***	-0·618 ***	-0·267 ***

© 2025 Chen M et al. *JAMA Network Open*.

	[-0·342, -0·306]	[-0·102, -0·045]	[-0·785, -0·733]	[-0·342, -0·290]	[-0·652, -0·584]	[-0·297, -0·237]
>= 80	-0·889 ***	-0·531 ***	-1·591 ***	-0·852 ***	-1·825 ***	-0·750 ***
	[-0·911, -0·867]	[-0·566, -0·495]	[-1·623, -1·559]	[-0·884, -0·820]	[-1·867, -1·783]	[-0·787, -0·713]
**Education**						
No education	Ref	Ref	Ref	Ref	Ref	Ref
Intermediate	0·268 ***	0·237 ***	0·499 ***	0·186 ***	0·430 ***	0·379 ***
	[0·252, 0·283]	[0·212, 0·262]	[0·476, 0·521]	[0·164, 0·209]	[0·401, 0·460]	[0·353, 0·405]
Higher education	0·476 ***	0·420 ***	0·938 ***	0·326 ***	0·710 ***	0·729 ***
	[0·458, 0·494]	[0·392, 0·449]	[0·912, 0·963]	[0·300, 0·352]	[0·676, 0·744]	[0·699, 0·758]
**Income**						
1st quartile	Ref	Ref	Ref	Ref	Ref	Ref
2nd quartile	0·138 ***	0·151 ***	0·187 ***	0·112 ***	0·273 ***	0·184 ***
	[0·121, 0·156]	[0·123, 0·179]	[0·162, 0·213]	[0·087, 0·138]	[0·239, 0·306]	[0·155, 0·213]
3rd quartile	0·205 ***	0·214 ***	0·270 ***	0·192 ***	0·387 ***	0·266 ***
	[0·187, 0·222]	[0·186, 0·242]	[0·244, 0·295]	[0·166, 0·217]	[0·354, 0·421]	[0·237, 0·296]
4th quartile	0·230 ***	0·219 ***	0·323 ***	0·239 ***	0·404 ***	0·314 ***
	[0·212, 0·248]	[0·189, 0·248]	[0·297, 0·349]	[0·212, 0·265]	[0·370, 0·439]	[0·284, 0·345]
**Country**						
Austria	Ref	Ref	Ref	Ref	Ref	Ref
Belgium	-0·113 ***	-0·378 ***	-0·333 ***	0·049 *	-0·030	-0·118 ***
	[-0·145, -0·081]	[-0·429, -0·327]	[-0·378, -0·287]	[0·003, 0·096]	[-0·090, 0·031]	[-0·171, -0·065]
Czech Republic	-0·135 ***	-0·202 ***	-0·279 ***	-0·289 ***	-0·020	-0·253 ***
	[-0·167, -0·104]	[-0·253, -0·151]	[-0·325, -0·233]	[-0·335, -0·243]	[-0·080, 0·040]	[-0·306, -0·200]
Denmark	0·238 ***	0·187 ***	-0·123 ***	0·669 ***	0·527 ***	0·322 ***
	[0·204, 0·272]	[0·133, 0·242]	[-0·172, -0·074]	[0·620, 0·718]	[0·463, 0·592]	[0·265, 0·378]
Estonia	-0·404 ***	-0·499 ***	-0·541 ***	-0·630 ***	-0·546 ***	-0·543 ***
	[-0·435, -0·373]	[-0·549, -0·449]	[-0·586, -0·496]	[-0·676, -0·585]	[-0·605, -0·487]	[-0·595, -0·491]
France	-0·122 ***	-0·508 ***	-0·435 ***	0·101 ***	0·036	-0·086 **
	[-0·157, -0·088]	[-0·562, -0·453]	[-0·485, -0·386]	[0·052, 0·151]	[-0·029, 0·101]	[-0·143, -0·029]
Germany	-0·074 ***	-0·286 ***	-0·363 ***	0·007	0·084 **	-0·037
	[-0·105, -0·042]	[-0·337, -0·235]	[-0·409, -0·318]	[-0·039, 0·053]	[0·024, 0·143]	[-0·089, 0·015]
Israel	-0·283 ***	-0·274 ***	-0·767 ***	-0·339 ***	-0·189 ***	-0·317 ***
	[-0·323, -0·243]	[-0·338, -0·210]	[-0·825, -0·710]	[-0·397, -0·281]	[-0·265, -0·114]	[-0·383, -0·251]
Italy	-0·419 ***	-0·753 ***	-0·893 ***	-0·613 ***	-0·405 ***	-0·315 ***
	[-0·453, -0·385]	[-0·807, -0·698]	[-0·942, -0·844]	[-0·663, -0·564]	[-0·470, -0·340]	[-0·371, -0·258]
Luxembourg	-0·053 *	-0·248 ***	-0·384 ***	0·168 ***	0·115 **	-0·073
	[-0·099, -0·007]	[-0·321, -0·175]	[-0·450, -0·318]	[0·101, 0·234]	[0·028, 0·202]	[-0·149, 0·003]
Netherlands	-0·014	-0·125 ***	-0·201 ***	-0·126 ***	0·185 ***	0·043
	[-0·048, 0·020]	[-0·179, -0·070]	[-0·250, -0·151]	[-0·175, -0·076]	[0·120, 0·249]	[-0·014, 0·100]
Slovenia	-0·265 ***	-0·293 ***	-0·600 ***	-0·313 ***	-0·264 ***	-0·374 ***
	[-0·303, -0·228]	[-0·353, -0·232]	[-0·654, -0·546]	[-0·368, -0·259]	[-0·335, -0·192]	[-0·436, -0·312]
Spain	-0·322 ***	-0·263 ***	-1·003 ***	-0·544 ***	-0·196 ***	-0·295 ***
	[-0·355, -0·289]	[-0·316, -0·210]	[-1·051, -0·955]	[-0·592, -0·496]	[-0·259, -0·133]	[-0·350, -0·239]
Sweden	0·124 ***	-0·102 ***	-0·210 ***	0·476 ***	0·429 ***	0·159 ***
	[0·091, 0·158]	[-0·155, -0·049]	[-0·258, -0·163]	[0·428, 0·524]	[0·366, 0·492]	[0·103, 0·214]
Switzerland	0·157 ***	-0·055	0·011	0·010	0·474 ***	0·241 ***
	[0·120, 0·194]	[-0·114, 0·004]	[-0·042, 0·065]	[-0·043, 0·064]	[0·404, 0·545]	[0·179, 0·302]
**Multimorbidity**						
0	Ref	Ref	Ref	Ref	Ref	Ref
1 or 2	-0·289 ***	-0·398 ***	-0·133 ***	-0·176 ***	-0·562 ***	-0·566 ***
	[-0·305, -0·273]	[-0·423, -0·372]	[-0·156, -0·110]	[-0·199, -0·153]	[-0·592, -0·532]	[-0·592, -0·539]
3 or more	-0·770 ***	-0·908 ***	-0·337 ***	-0·485 ***	-1·642 ***	-1·330 ***
	[-0·789, -0·751]	[-0·938, -0·877]	[-0·364, -0·310]	[-0·512, -0·458]	[-1·678, -1·606]	[-1·361, -1·299]
**R^2**	0·365	0·174	0·359	0·216	0·374	0·268
*** p < 0·001; ** p < 0·01; * p < 0·05.						

© 2025 Chen M et al. *JAMA Network Open*.

Notes: *** p < 0.001; ** p < 0.01; * p < 0.05. Sample size N = 64 872. Missing value percentage: Education (6.3%), Income (1.0%), and Multimorbidity (0.1%). All the other variables are complete. Controlled for country. Austria was selected as the reference country based on an alphabetically sorted list.

© 2025 Chen M et al. *JAMA Network Open*.

eTable 3. Comparison of Sample Characteristics Between Participants Included in the Mediation Analysis and Those Lost to Follow-Up Between Wave 5 and Wave 6

a. Mediation analysis for ADL (N = 41 641).

Characteristics		Mediation analysis	Lost to follow-up
**Women (%)**		55.77	52.70
**Age (SD)**		66.29 (9.35)	66.04 (9.98)
**Education (%)**	Lower	33.92	37.78
	Intermediate	40.60	37.42
	Higher	25.48	24.80
**Multimorbidity (%)**	0	24.10	26.70
	1 or 2	52.73	51.38
	3 or more	23.17	21.92

b. Mediation analysis for IADL (N = 39 171).

Characteristics		Mediation analysis	Lost to follow-up
**Women (%)**		54.22	51.02
**Age (SD)**		65.81 (9.06)	65.32 (9.51)
**Education (%)**	Lower	32.47	36.50
	Intermediate	41.32	38.11
	Higher	26.21	25.38
**Multimorbidity (%)**	0	25.23	28.38
	1 or 2	53.35	51.80
	3 or more	21.42	19.82

Notes: ADL: Activities of Daily Living; IADL: Instrumental Activities of Daily Living.

© 2025 Chen M et al. *JAMA Network Open*.

eTable 4. Regression Coefficients for the Direct Effects of IC on ADLs and IADLs, and Indirect Effects Through Multimorbidity

**Causal path**	**ADLs (N = 41 641)**		**IADLs (N = 39 171)**	
	**Standardised coefficient**	**p-value**	**Standardised coefficient**	**p-value**
**Total effect**				
Intrinsic capacity	-0.155	0.000	-0.239	0.000
**Direct effect**				
Intrinsic capacity	-0.148	0.000	-0.231	0.000
**Indirect effect**				
Intrinsic capacity → multimorbidity	-0.007	0.000	-0.007	0.000

Note: Controlled for age, gender, education, income, and country.

eFigure 2. Direct and Indirect Effects of Personal Characteristics on ADL

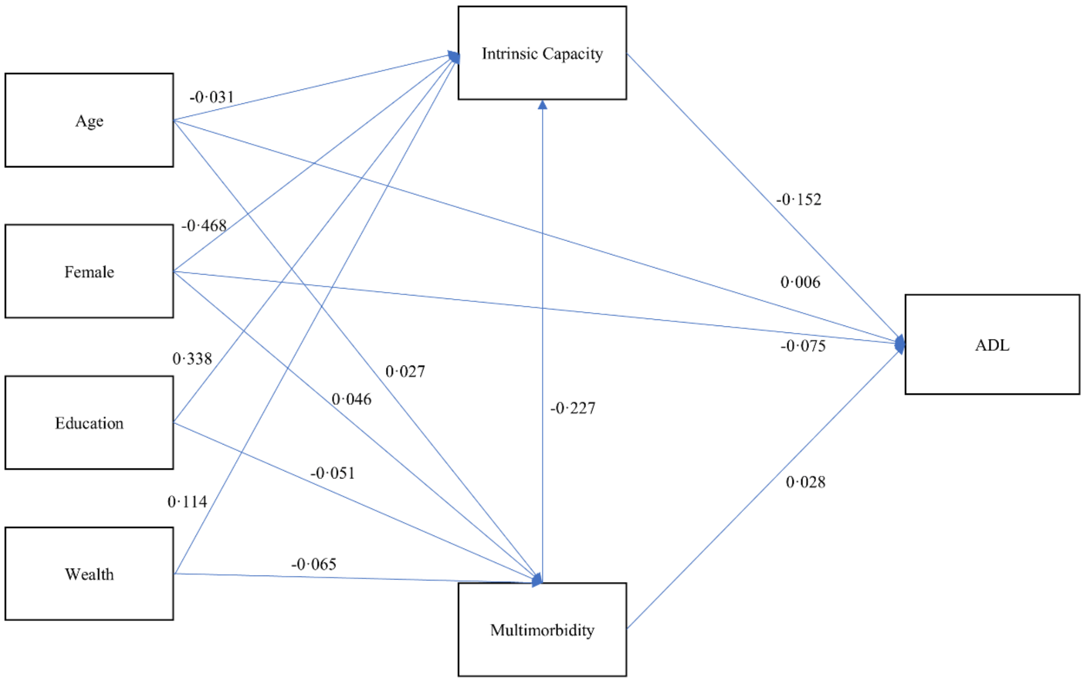

Note: ADL denotes activities of daily living.

eFigure 3. Direct and Indirect Effects of Personal Characteristics on IADL

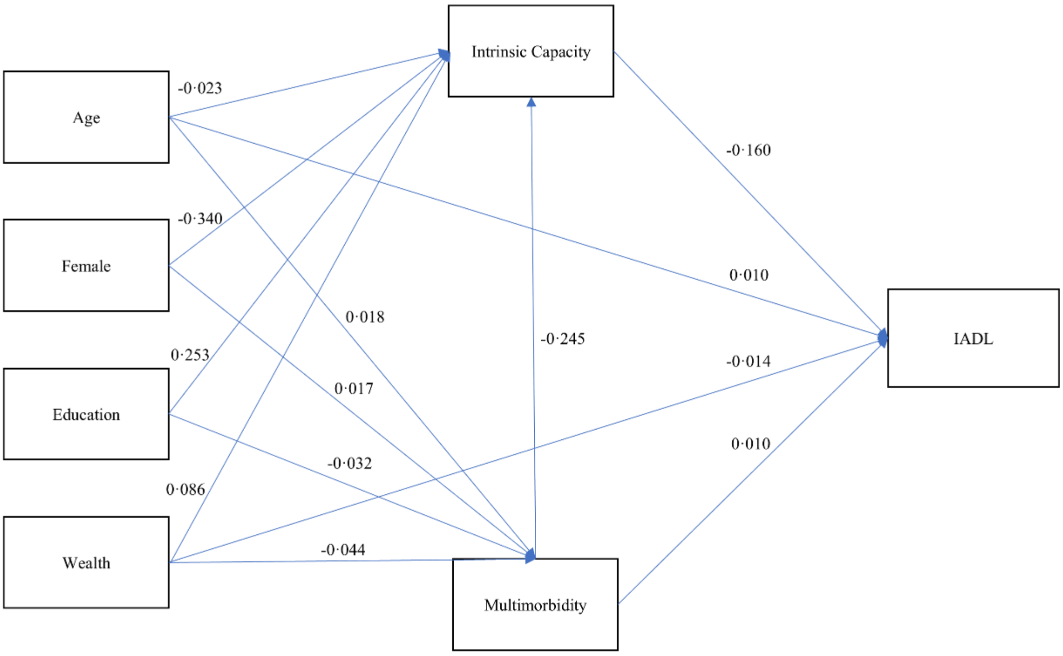

Note: IADL denotes instrumental activities of daily living.

© 2025 Chen M et al. *JAMA Network Open*.

eTable 5. Direct and Indirect Effects of Personal Characteristics on ADL and IADL Through Mediators (IC and Multimorbidity)

	**ADLs (N = 41 641)**		**IADLs (N = 39 171)**	
**Causal Path**	**Standardised coefficient (SE)**	**p-value**	**Standardised coefficient (SE)**	**p-value**
**Direct effect**				
Age	0.095 (0.000)	0.000	0.148 (0.000)	0.000
Gender (women)	-0.007 (0.002)	0.132	0.044 (0.003)	0.000
Education	0.004 (0.002)	0.476	-0.012 (0.002)	0.024
Income	-0.010 (0.001)	0.035	-0.000 (0.002)	0.999
Multimorbidity	0.039 (0.002)	0.000	0.041 (0.002)	0.000
Intrinsic capacity	-0.213 (0.002)	0.000	-0.209 (0.002)	0.000
**Specific indirect effect**				
Age → IC	0.056 (0.000)	0.000	0.050 (0.000)	0.000
Gender → IC	0.016 (0.001)	0.000	0.011 (0.001)	0.000
Education → IC	-0.049 (0.001)	0.000	-0.047 (0.001)	0.000
Income → IC	-0.016 (0.000)	0.000	-0.015 (0.000)	0.000
Age → multimorbidity	0.010 (0.000)	0.000	0.010 (0.000)	0.000
Gender → multimorbidity	0.001 (0.000)	0.000	0.001 (0.000)	0.000
Education → multimorbidity	-0.002 (0.000)	0.000	-0.002 (0.000)	0.000
Income → multimorbidity	-0.002 (0.000)	0.000	-0.002 (0.000)	0.000
**Indirect effect**				
Age → multimorbidity → IC	0.015 (0.000)	0.000	0.014 (0.000)	0.000
Gender → multimorbidity → IC	0.002 (0.000)	0.000	0.001 (0.000)	0.000
Education → multimorbidity → IC	-0.003 (0.000)	0.000	-0.003 (0.000)	0.000
Income → multimorbidity → IC	-0.003 (0.000)	0.000	-0.003 (0.000)	0.000

Notes: Adjusted for countries through two-level serial mediation analysis. SE denotes standard error.

© 2025 Chen M et al. *JAMA Network Open*.

Analysis of IC values

eTable 6. Mean Values and Standard Deviations of IC and Subdomains Across Genders and Countries

Country	gender	IC (SD)	Psychological (SD)	Sensory (SD)	Cognitive (SD)	Locomotor (SD)	Vitality (SD)
**Austria**	Women	60.631 (10.978)	64.841 (17.703)	60.886 (16.566)	65.566 (17.849)	62.161 (22.070)	61.966 (20.219)
	Men	62.505 (10.408)	69.777 (18.033)	61.820 (15.713)	63.957 (16.583)	67.705 (19.416)	63.042 (18.630)
**Belgium**	Women	58.077 (11.356)	58.562 (19.815)	60.638 (17.616)	60.469 (18.451)	59.162 (22.752)	58.652 (20.593)
	Men	60.658 (10.366)	63.187 (20.013)	61.409 (17.118)	60.613 (17.866)	66.682 (18.944)	61.190 (18.924)
**Czech Republic**	Women	57.333 (11.402)	59.619 (18.983)	55.160 (18.263)	61.776 (17.343)	59.576 (22.473)	55.331 (19.982)
	Men	60.03 (10.021)	65.547 (18.639)	56.882 (17.6)	60.516 (15.851)	66.413 (19.453)	57.521 (18.236)
**Denmark**	Women	63.448 (10.998)	66.876 (18.665)	69.687 (18.466)	65.192 (17.492)	67.833 (20.818)	64.932 (20.919)
	Men	65.052 (9.733)	70.945 (18.435)	71.188 (17.467)	63.004 (16.31)	72.762 (17.551)	66.704 (18.383)
**Estonia**	Women	55.087 (11.26)	58.042 (17.278)	51.682 (17.904)	59.828 (18.36)	55.000 (23.807)	52.637 (19.526)
	Men	56.968 (10.158)	60.695 (17.444)	50.788 (16.851)	56.400 (17.212)	60.721 (20.833)	57.542 (18.016)
**France**	Women	56.923 (11.868)	54.906 (19.756)	60.021 (19.589)	57.737 (19.278)	58.689 (23.184)	57.772 (21.009)
	Men	59.781 (10.596)	61.315 (19.578)	62.037 (18.985)	56.874 (17.351)	66.479 (19.192)	60.889 (18.477)
**Germany**	Women	58.317 (11.211)	58.928 (18.318)	59.032 (17.637)	60.798 (18.079)	60.197 (22.356)	59.866 (20.303)
	Men	60.17 (10.507)	63.785 (19.027)	60.463 (17.726)	58.907 (17.029)	65.653 (19.710)	60.550 (18.827)
**Israel**	Women	57.787 (12.099)	62.683 (20.385)	56.829 (17.425)	56.667 (17.333)	60.461 (23.871)	58.040 (19.384)
	Men	60.722 (10.811)	67.770 (19.682)	59.014 (17.409)	57.217 (16.395)	67.789 (19.591)	60.899 (18.240)
**Italy**	Women	52.786 (11.814)	52.253 (20.964)	49.766 (18.634)	49.534 (18.193)	53.384 (22.052)	54.813 (20.312)
	Men	56.374 (10.54)	58.654 (20.178)	52.046 (17.975)	51.328 (17.35)	61.870 (17.840)	57.893 (18.117)
**Luxembourg**	Women	57.238 (11.451)	57.848 (18.856)	60.790 (17.998)	58.731 (18.687)	57.834 (22.063)	56.867 (20.940)
	Men	58.836 (10.867)	62.462 (19.124)	61.621 (17.08)	56.699 (17.76)	64.581 (19.738)	56.410 (20.890)
**Netherlands**	Women	58.618 (10.588)	60.789 (19.351)	56.929 (17.197)	61.792 (17.042)	61.381 (21.022)	59.375 (20.724)
	Men	61.523 (9.385)	65.076 (20.508)	59.336 (17.146)	59.928 (16.798)	68.458 (16.605)	62.877 (16.709)
**Slovenia**	Women	56.031 (11.362)	59.437 (17.592)	55.063 (16.813)	55.521 (18.798)	57.169 (23.502)	55.278 (19.868)
	Men	59.125 (10.322)	64.979 (17.061)	57.859 (16.596)	56.688 (18.174)	65.822 (20.324)	57.353 (17.934)
**Spain**	Women	53.541 (11.773)	57.529 (21.435)	51.272 (16.528)	48.402 (18.643)	55.707 (22.715)	52.167 (19.775)
	Men	56.150 (10.700)	65.070 (19.314)	51.053 (15.877)	48.091 (18.097)	61.843 (19.858)	55.428 (17.881)
**Sweden**	Women	60.563 (10.775)	61.141 (18.050)	65.661 (20.139)	62.615 (17.479)	63.651 (20.272)	62.029 (20.228)
	Men	62.090 (9.897)	65.611 (18.659)	66.997 (18.602)	58.852 (16.479)	69.698 (17.190)	61.799 (19.204)
**Switzerland**	Women	61.739	63.747	58.750	65.226	67.216	64.790

© 2025 Chen M et al. *JAMA Network Open*.

		(10.187)	(17.184)	(16.258)	(17.391)	(18.798)	(19.675)
	Men	64.054 (9.291)	68.555 (18.021)	61.484 (16.315)	64.251 (16.643)	72.990 (15.429)	66.560 (17.749)

Note: SD: standard deviation. Scores are weighted using calibrated weights.

© 2025 Chen M et al. *JAMA Network Open*.

eFigure 4. Mean Values of IC and Subdomains Scores for Different Genders and Socioeconomic Status Groups

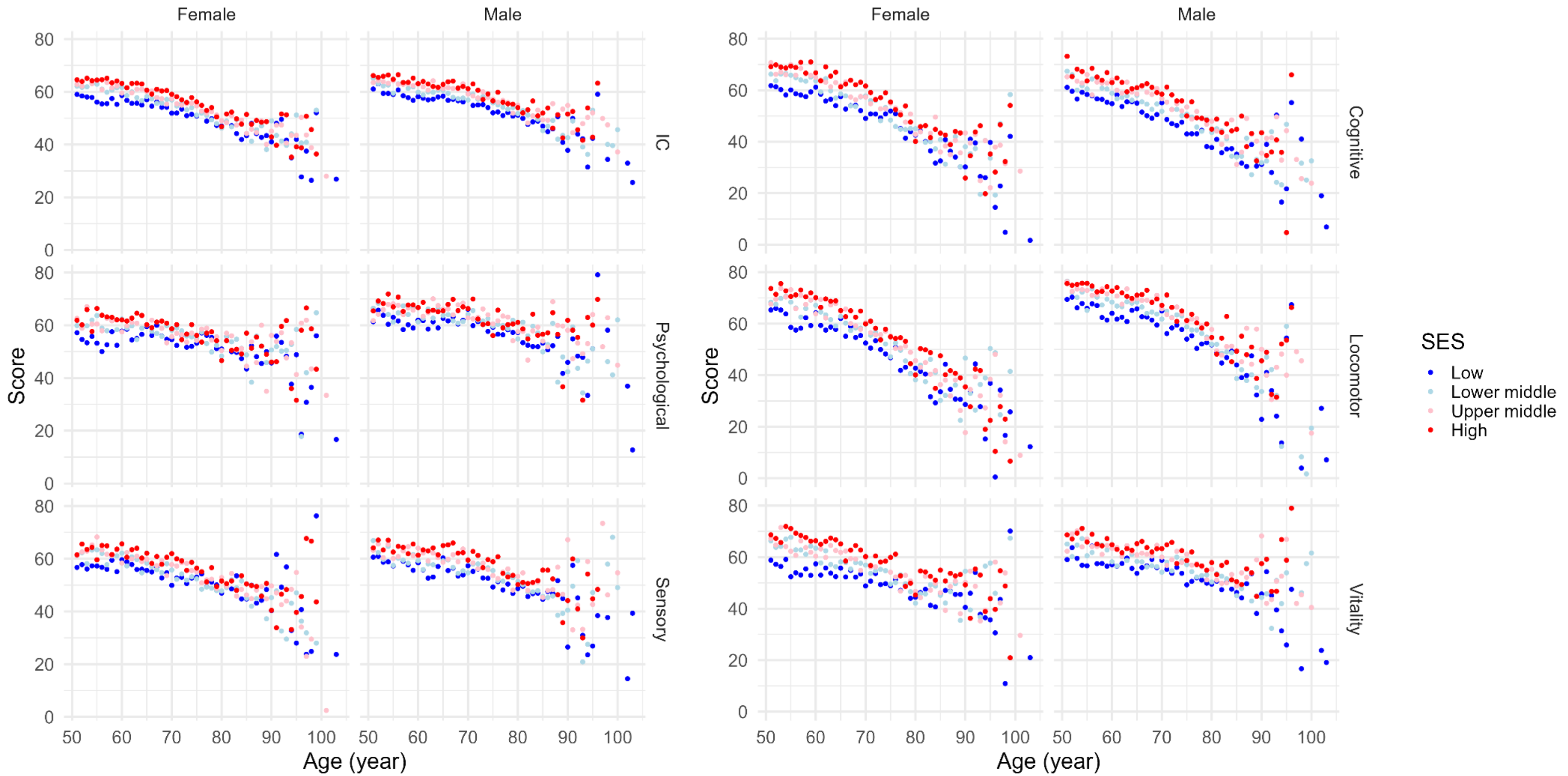

Note: SES is an abbreviation of socio-economic status.

© 2025 Chen M et al. *JAMA Network Open*.

eTable 7. Mean Value of IC and Subdomains for Participants of Different Ages, Socioeconomic Statuses, and Genders

Gender	Age	SES	IC	Psychological	Sensory	Cognitive	Locomotor	Vitality
Women	70-74	1st quartile	50.748	56.617	52.793	45.938	63.109	56.069
		2nd quartile	53.901	60.264	54.376	49.155	66.353	59.286
		3rd quartile	55.720	59.798	58.657	49.956	68.417	61.953
		4th quartile	57.074	60.836	60.206	52.457	68.467	62.247
	75-79	1st quartile	46.077	55.128	50.000	41.163	55.924	52.618
		2nd quartile	48.474	56.483	50.184	42.570	59.644	54.978
		3rd quartile	50.860	58.365	52.918	46.507	62.840	55.705
		4th quartile	50.573	56.770	53.194	45.636	61.650	57.320
	80-84	1st quartile	40.109	50.821	44.959	34.681	47.982	48.529
		2nd quartile	42.559	52.767	47.150	36.707	51.478	49.899
		3rd quartile	44.888	54.191	48.587	40.284	54.394	51.625
		4th quartile	46.062	52.760	50.146	39.045	56.175	54.625
	85-89	1st quartile	36.305	46.810	40.714	30.710	43.109	47.454
		2nd quartile	37.862	49.758	41.620	33.843	44.110	48.216
		3rd quartile	40.055	49.602	45.439	32.758	47.563	50.556
		4th quartile	42.449	52.209	46.454	35.946	50.657	52.751
	>=90	1st quartile	32.826	43.900	37.656	26.608	38.791	44.846
		2nd quartile	33.035	45.416	35.081	27.987	38.985	47.914
		3rd quartile	39.045	51.366	43.504	33.627	42.930	51.431
		4th quartile	36.481	47.883	39.304	30.023	42.494	48.475
Men	70-74	1st quartile	54.251	62.387	55.202	44.794	68.154	59.415
		2nd quartile	57.914	65.614	58.305	48.289	72.487	61.891
		3rd quartile	58.652	65.871	58.976	49.510	73.259	63.199
		4th quartile	60.505	66.626	61.110	52.257	74.663	65.783
	75-79	1st quartile	50.146	59.621	50.418	40.011	63.696	56.043
		2nd quartile	54.104	62.574	54.401	44.790	67.993	59.147
		3rd quartile	56.176	63.817	54.896	46.658	71.090	61.915
		4th quartile	56.539	63.347	57.476	46.970	70.554	62.021
	80-84	1st quartile	46.388	55.401	47.782	36.093	58.979	54.030
		2nd quartile	47.987	58.241	48.472	38.445	59.460	55.654
		3rd quartile	50.103	57.961	52.018	41.013	63.360	56.331
		4th quartile	51.665	60.838	51.608	42.013	64.079	58.860
	85-89	1st quartile	41.461	52.037	45.167	31.792	51.889	51.072
		2nd quartile	42.059	52.091	42.817	33.573	52.843	51.160
		3rd quartile	48.128	57.393	47.349	36.186	60.168	56.322
		4th quartile	50.289	58.136	52.823	41.164	63.926	57.076
	>=90	1st quartile	36.968	48.162	39.803	30.391	43.915	48.331
		2nd quartile	34.365	44.139	34.614	26.969	41.669	47.568
		3rd quartile	42.598	51.567	42.717	36.229	49.545	54.858
		4th quartile	45.777	56.947	45.840	36.512	53.934	57.055

Note: 1st quartile denotes the lowest socio-economic status, while 4th quartile denotes the highest.

© 2025 Chen M et al. *JAMA Network Open*.

eFigure 5. Centile Curves for Subdomains

a). Centile curves for psychological subdomain.

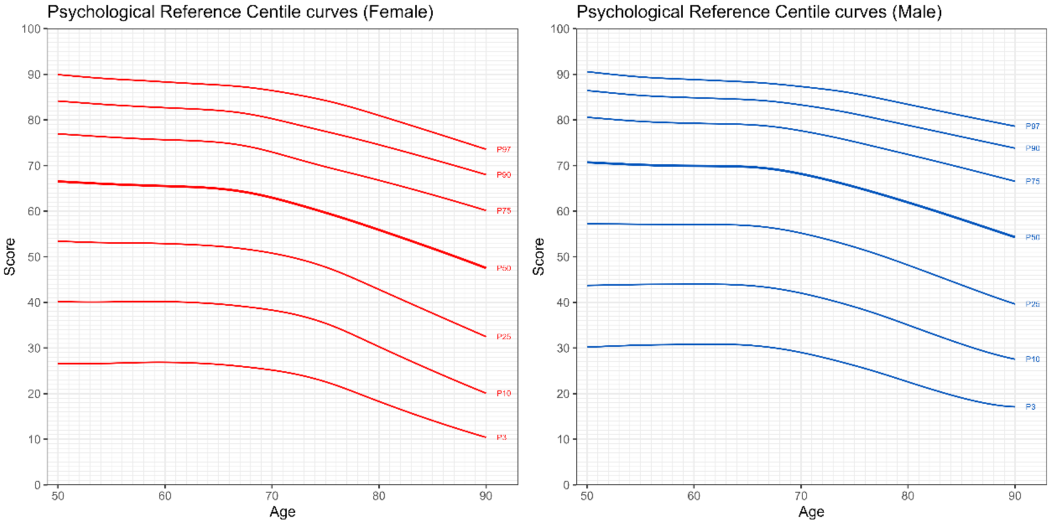

b). Centile curves for sensory subdomain.

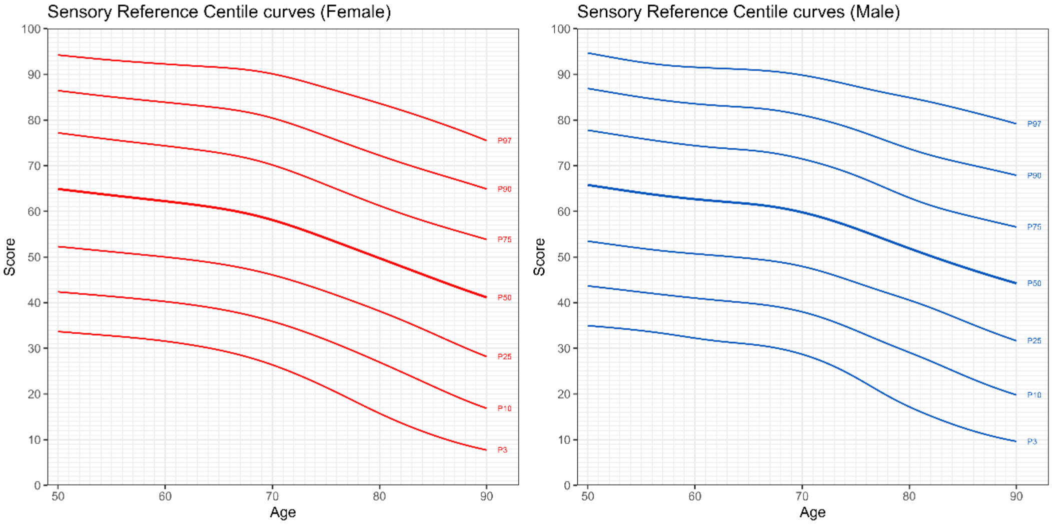

c). Centile curves for cognitive subdomain.

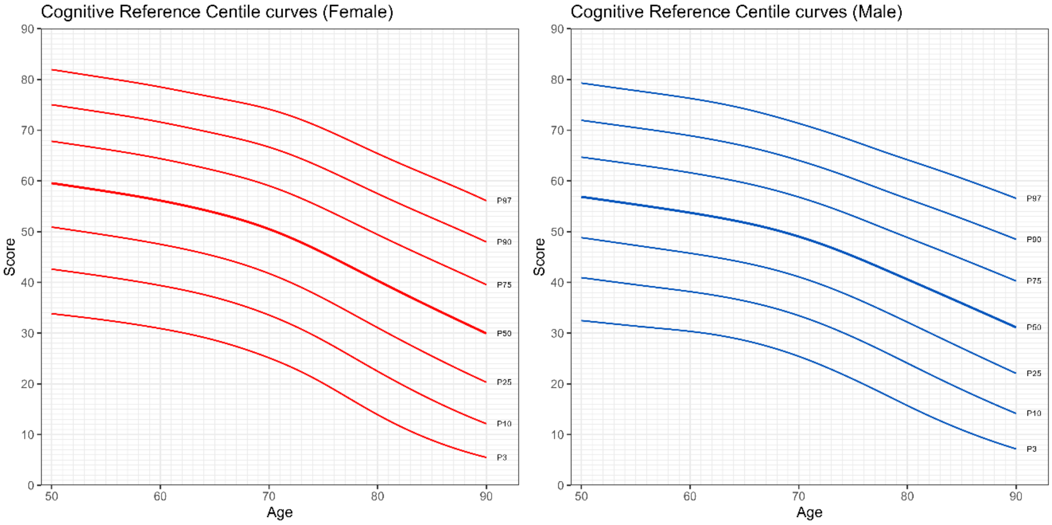

© 2025 Chen M et al. *JAMA Network Open*.

d). Centile curves for locomotor subdomain.

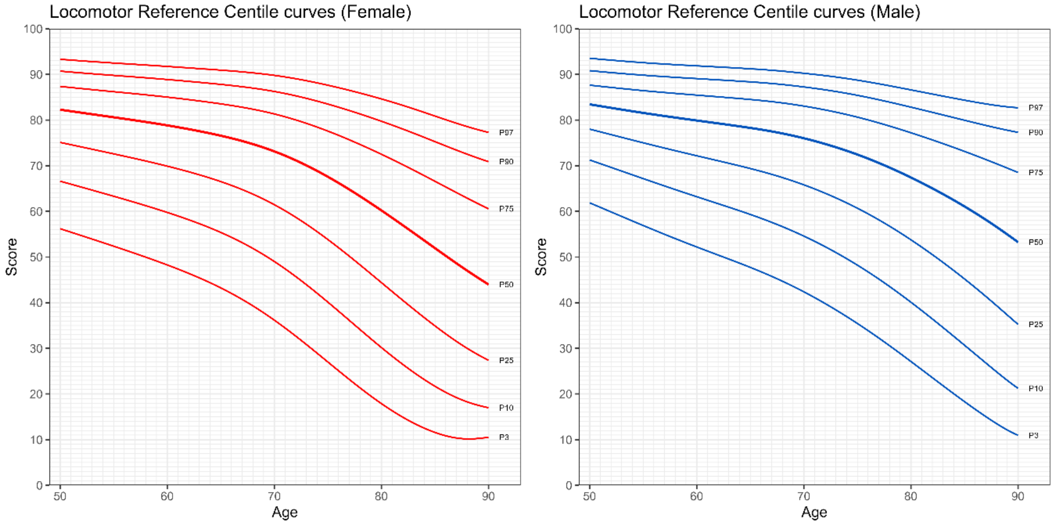

e). Centile curves for vitality subdomain.

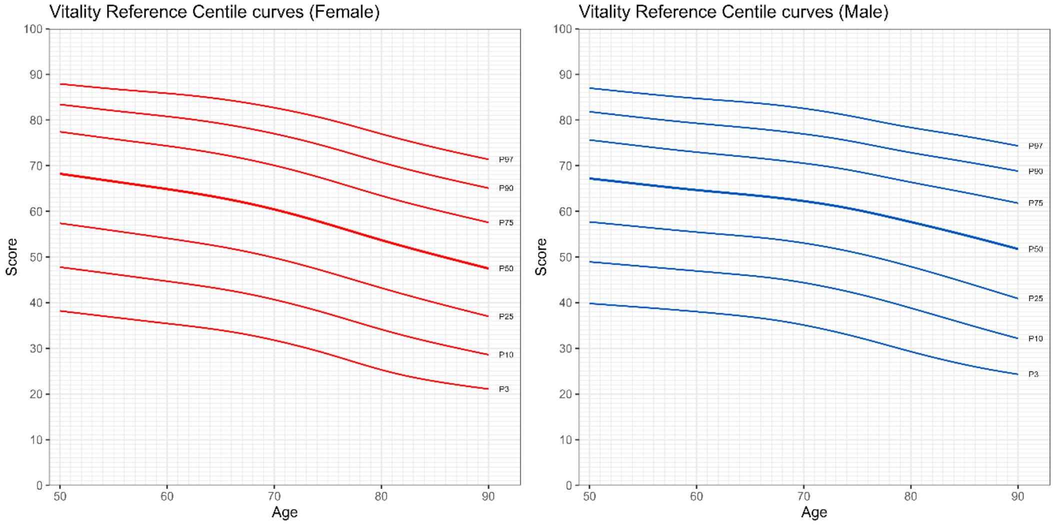

© 2025 Chen M et al. *JAMA Network Open*.

eFigure 6. Centile Curves for IC in Different Countries

a). Austria

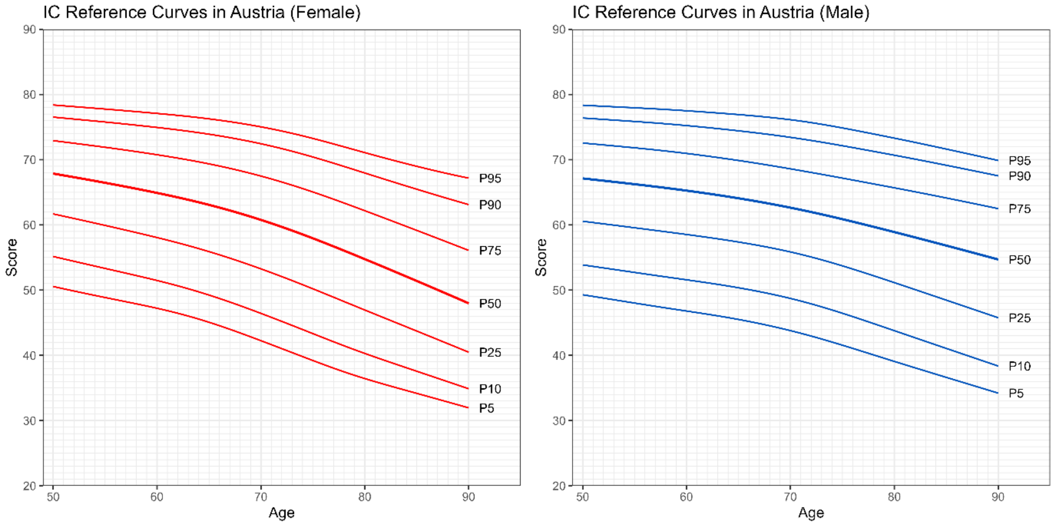

b). Belgium

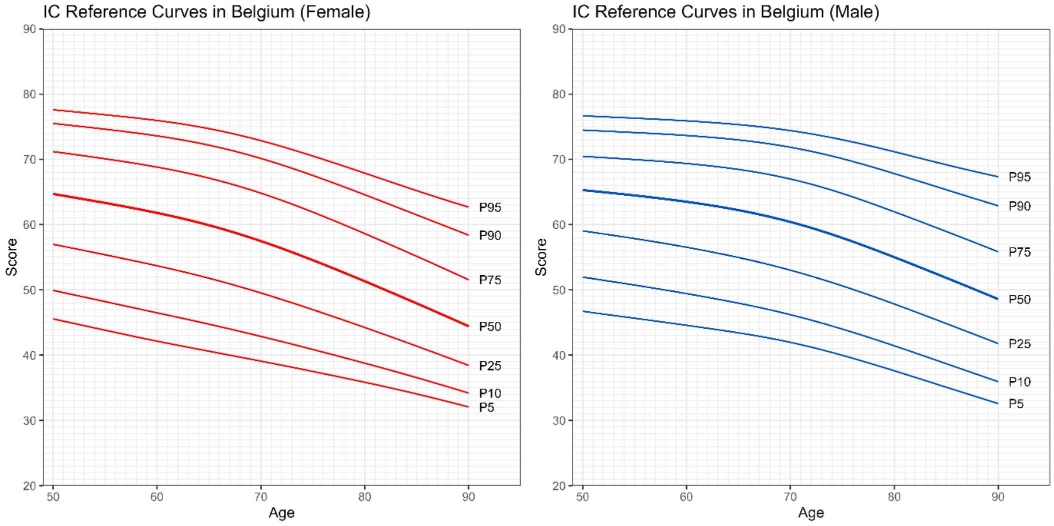

c). Czech Republic

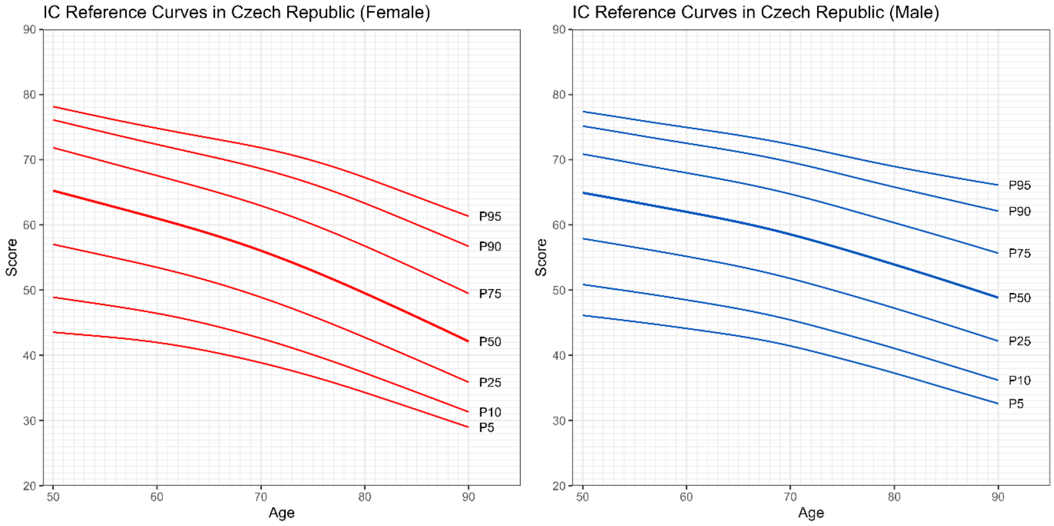

© 2025 Chen M et al. *JAMA Network Open*.

d). Denmark

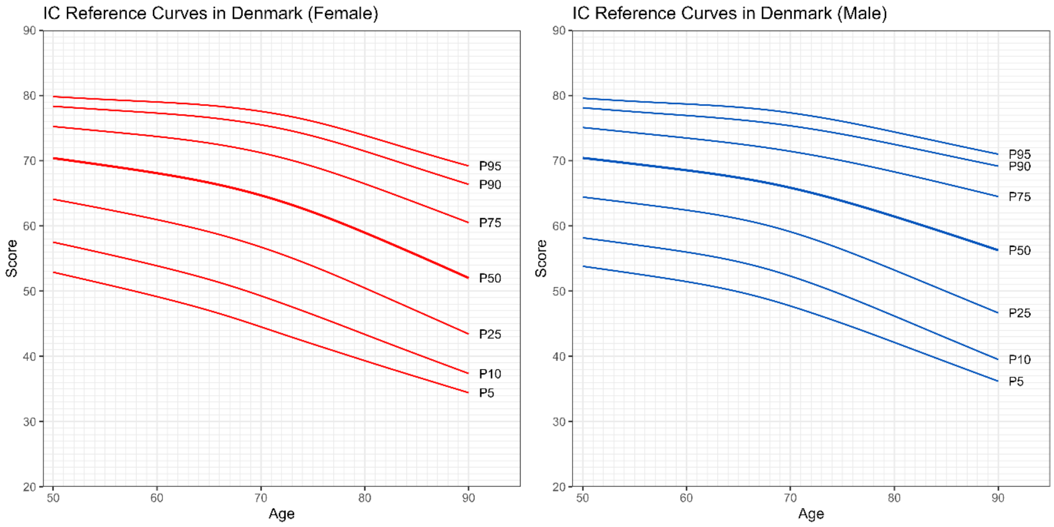

e). Estonia

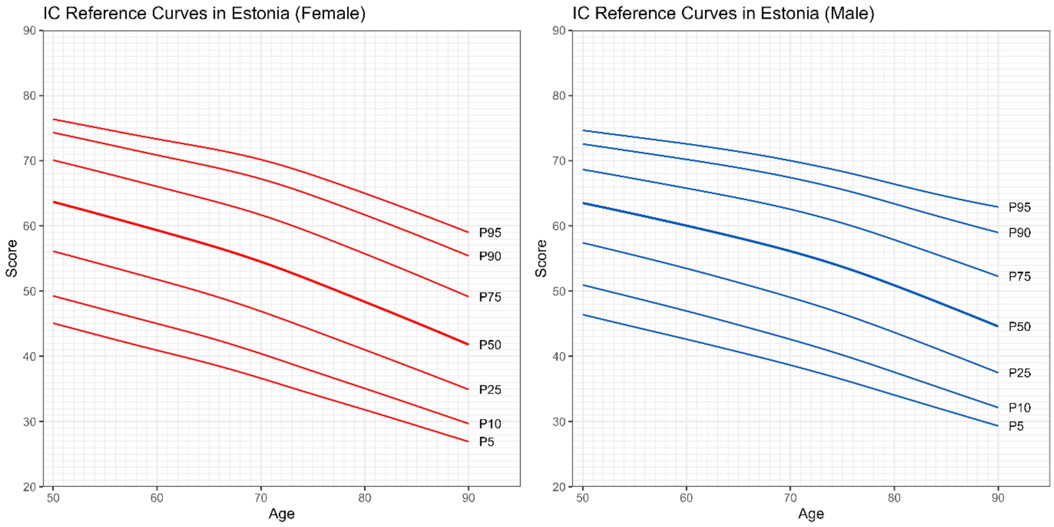

f). France

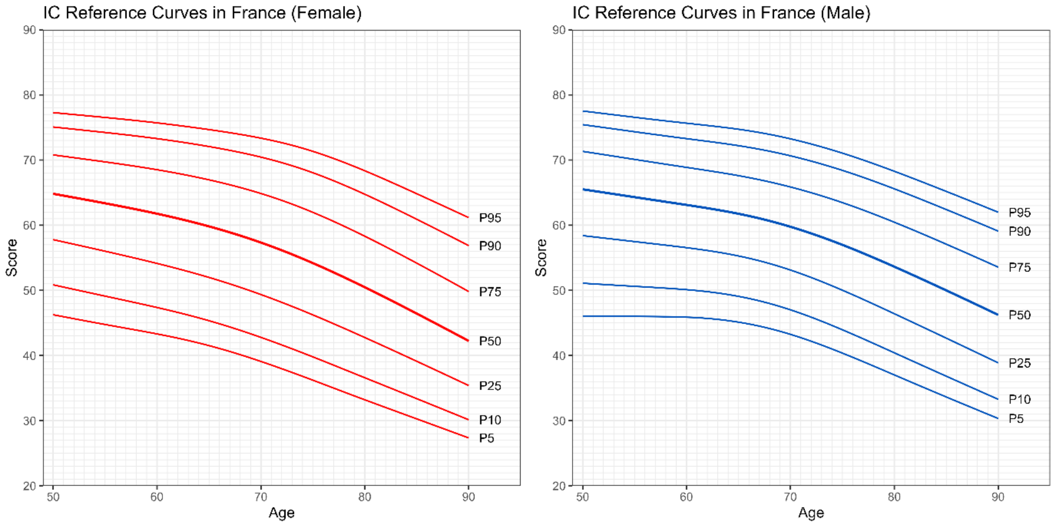

© 2025 Chen M et al. *JAMA Network Open*.

g). Germany

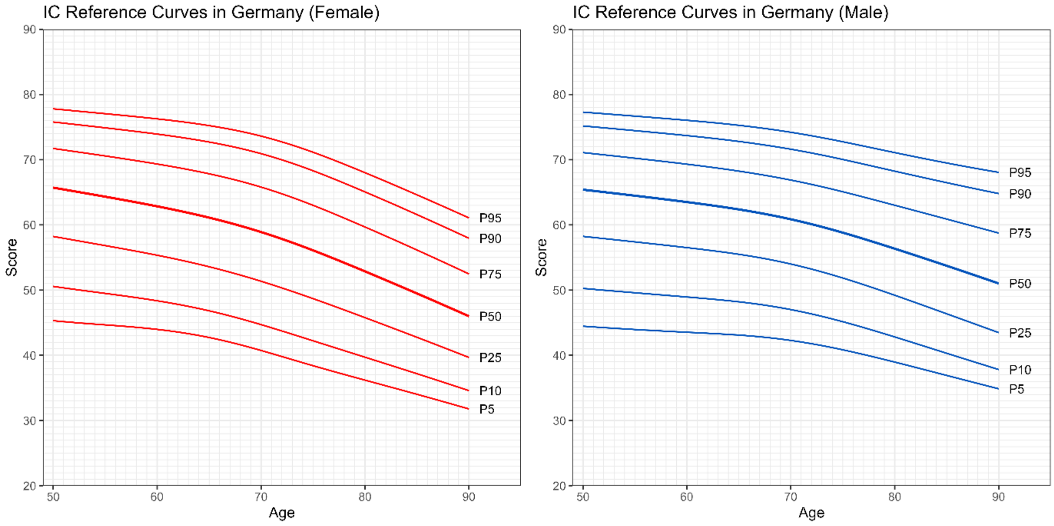

h). Israel

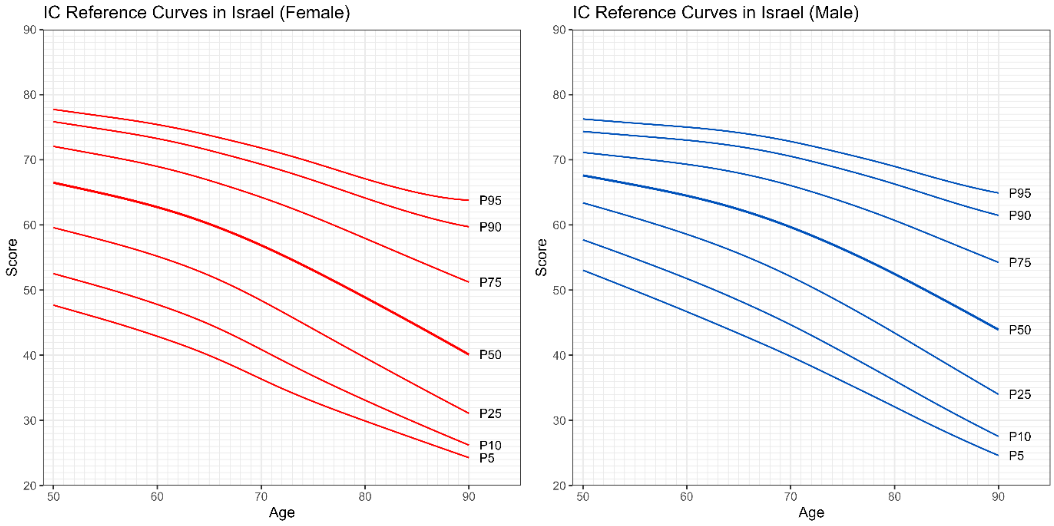

i). Italy

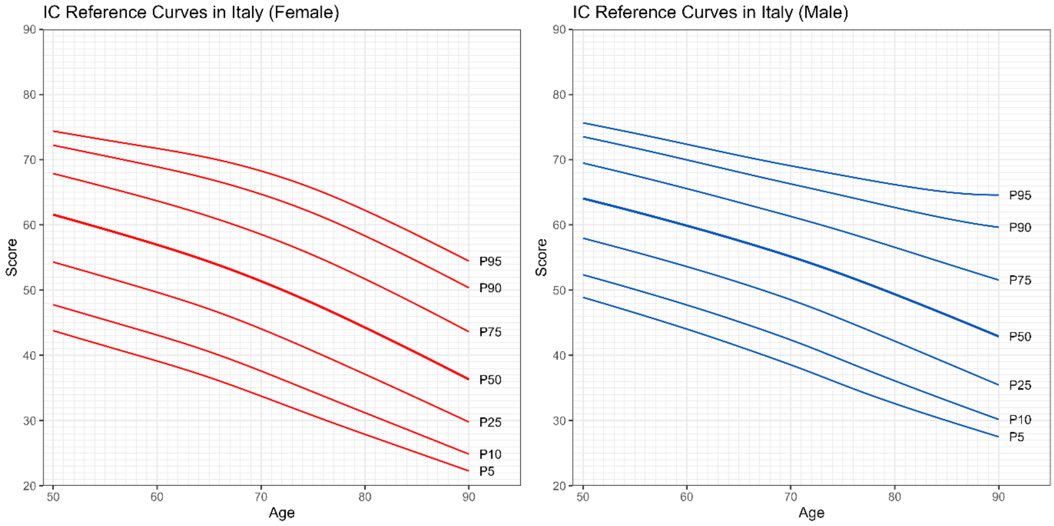

© 2025 Chen M et al. *JAMA Network Open*.

j). Luxembourg

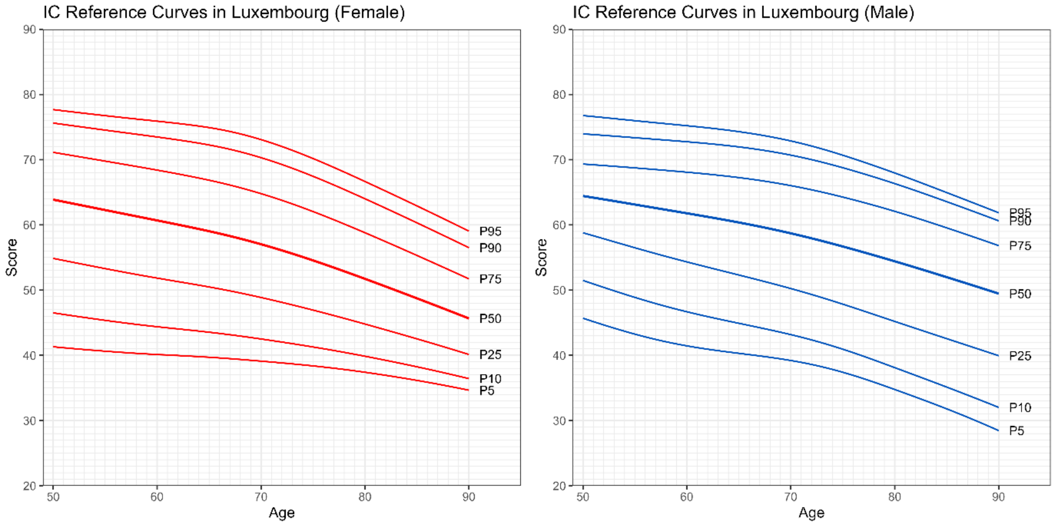

k). Netherlands

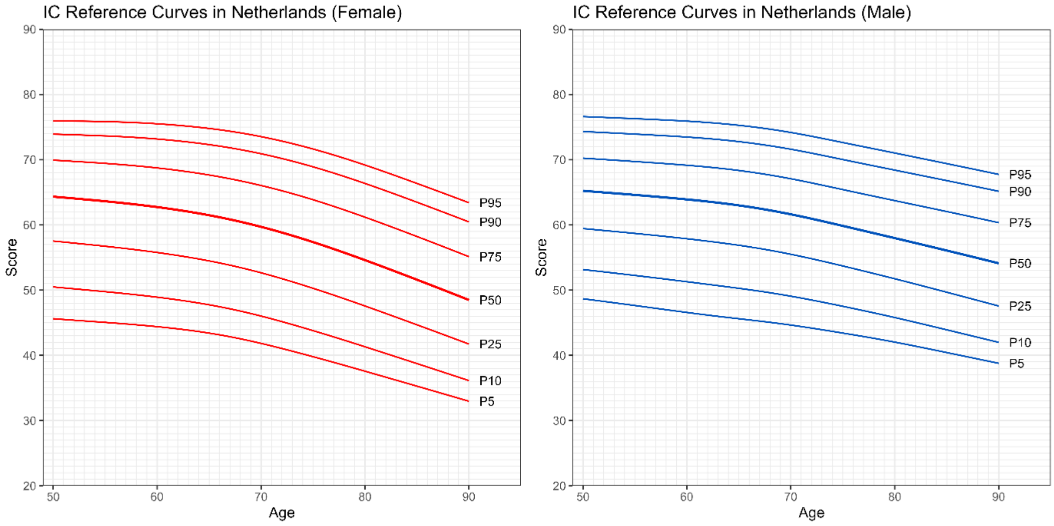

l). Slovenia

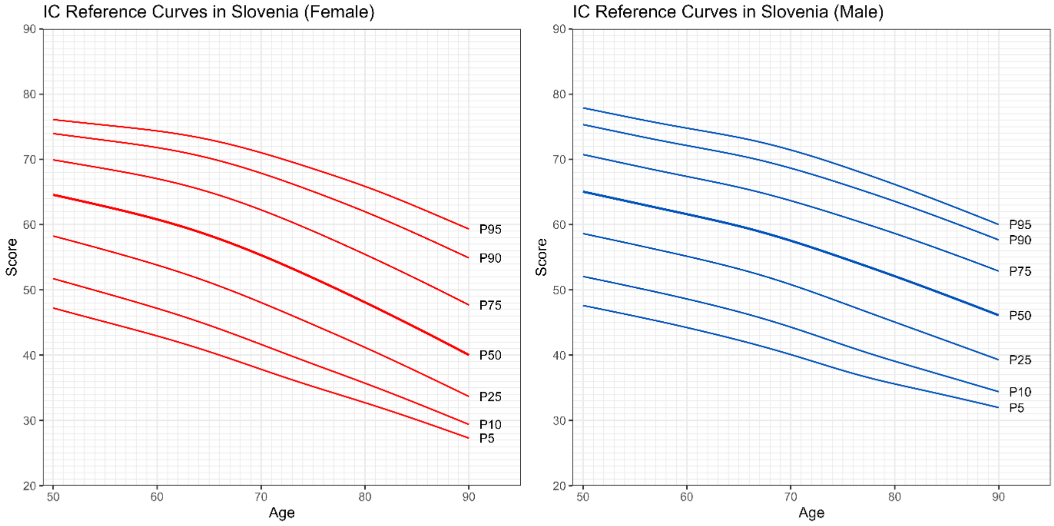

© 2025 Chen M et al. *JAMA Network Open*.

m). Spain

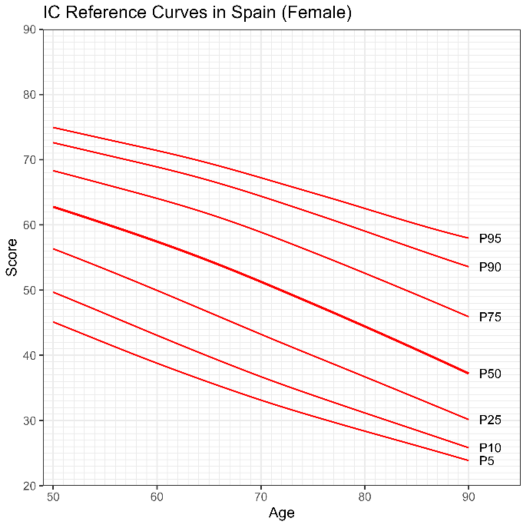

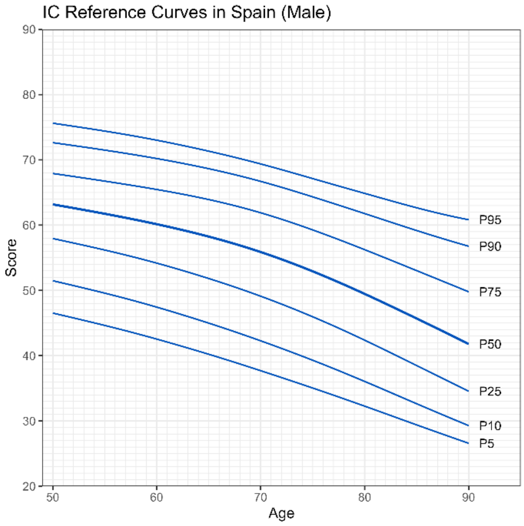

n). Sweden

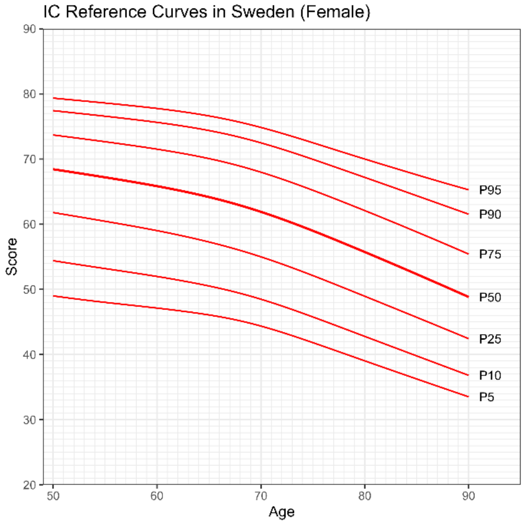

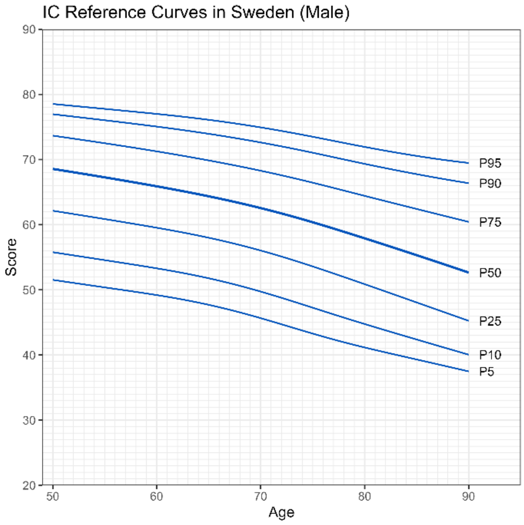

o). Switzerland

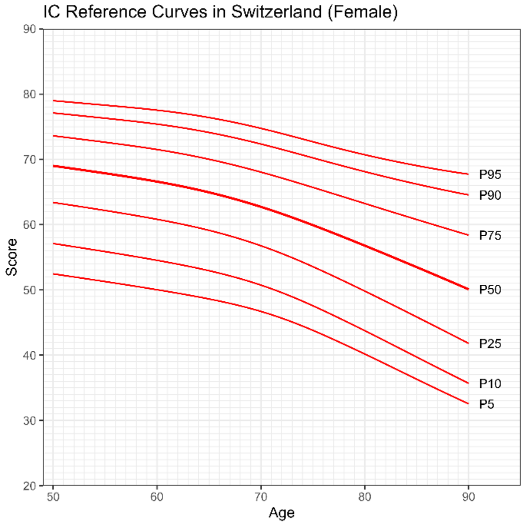

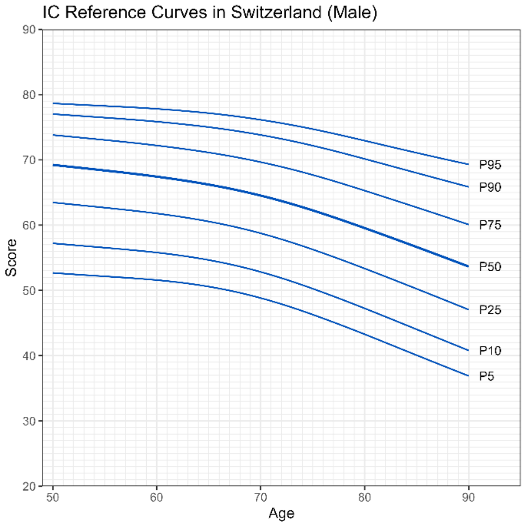

© 2025 Chen M et al. *JAMA Network Open*.

eFigure 7. Intrinsic Capacity and Subdomain Scores Across Different Countries for Men

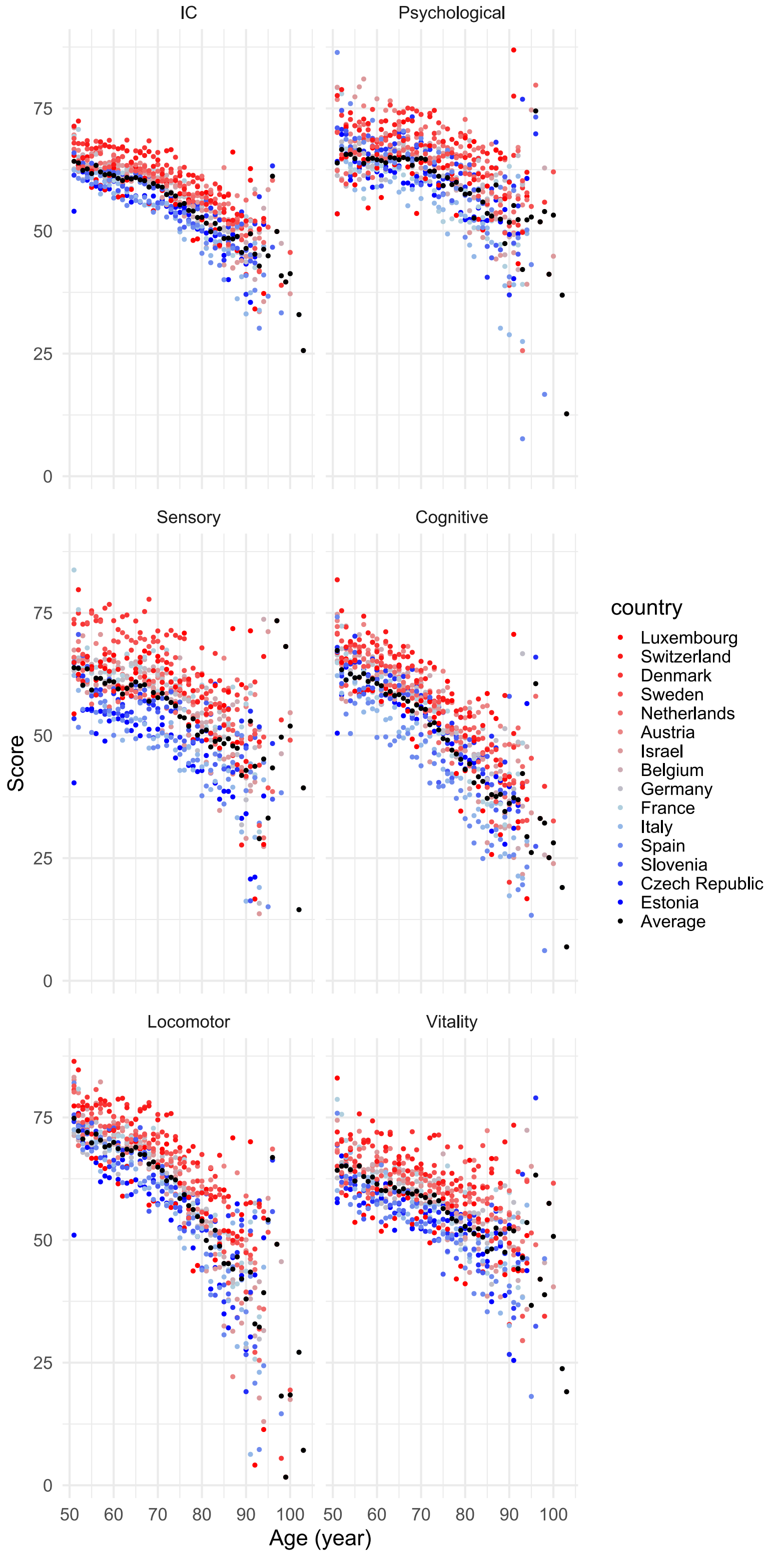

Note: Each point represents the mean score for IC or its subdomains for participants of a certain age across different countries. Black points indicate the overall mean values across all the countries. Countries are ordered by 2013 gross domestic product (GDP) per capita: those with a higher GDP per capita are depicted in red, while those with a lower GDP per capita are depicted in blue. GDP per capita data for the studied countries were retrieved from World Bank Group.

© 2025 Chen M et al. *JAMA Network Open*.

eReferences.

1 Börsch-Supan A, Brandt M, Hunkler C, *et al.* Data Resource Profile: The Survey of Health, Ageing and Retirement in Europe (SHARE). *Int J Epidemiol* 2013; **42**: 992–1001.

2 Prince MJ, Reischies F, Beekman ATF, *et al.* Development of the EURO–D scale – a European Union initiative to compare symptoms of depression in 14 European centres. *Br J Psychiatry* 1999; **174**: 330–8.

3 Deville J-C, Särndal C-E. Calibration Estimators in Survey Sampling. *J Am Stat Assoc* 1992; **87**: 376–82.

4 Cole TJ, Freeman JV, Preece MA. Body mass index reference curves for the UK, 1990. *Arch Dis Child* 1995; **73**: 25–9.

5 Eilers PHC, Marx BD, Durban M. Twenty years of P-splines. *SORT Stat Oper Res Trans* 2015; **39**: 0149-- 186.

© 2025 Chen M et al. *JAMA Network Open*.
