# Supplementary material for: Intrinsic Capacity Across 15 Countries in the Survey of Health, Aging, and Retirement in Europe
Source: JAMA Netw Open. 2025 May 12;8(5):e259792. doi: 10.1001/jamanetworkopen.2025.9792 (PMC12070240; doi:10.1001/jamanetworkopen.2025.9792)
Supplement: Supplement 2. — Data Sharing Statement [file jamanetwopen-e259792-s002.pdf]

Data Sharing Statement

Chen. Intrinsic Capacity Across 15 Countries in the Survey of Health, Aging, and Retirement in Europe. *JAMA Netw Open*. Published May 12, 2025. doi:10.1001/jamanetworkopen.2025.9792

Data

Data available: No

Additional Information

Explanation for why data not available: The data used in this study are available upon registration on the SHARE website (https://www.share-eric.eu/).
